# Distribution and molecular evolution of the anti-CRISPR family AcrIF7

**DOI:** 10.1371/journal.pbio.3002072

**Published:** 2023-04-21

**Authors:** Wendy Figueroa, Adrian Cazares, Daniel Cazares, Yi Wu, Ana de la Cruz, Martin Welch, Luis Kameyama, Franklin L. Nobrega, Gabriel Guarneros

**Affiliations:** 1 Department of Biochemistry, University of Cambridge, Cambridge, United Kingdom; 2 EMBL’s European Bioinformatics Institute (EMBL-EBI), Wellcome Genome Campus, Hinxton, United Kingdom; 3 Wellcome Sanger Institute, Wellcome Genome Campus, Hinxton, United Kingdom; 4 Centro de Ciencias Genómicas, Universidad Nacional Autónoma de México, Cuernavaca, Mexico; 5 School of Biological Sciences, Faculty of Environmental and Life Sciences, University of Southampton, Southampton, United Kingdom; 6 Department of Genetics and Molecular Biology, Center for Research and Advanced Studies of the National Polytechnic Institute, Mexico City, Mexico; Monash University, AUSTRALIA

## Abstract

Anti-clustered regularly interspaced short palindromic repeats (CRISPRs) are proteins capable of blocking CRISPR-Cas systems and typically their genes are located on mobile genetic elements. Since their discovery, numerous anti-CRISPR families have been identified. However, little is known about the distribution and sequence diversity of members within a family, nor how these traits influence the anti-CRISPR’s function and evolution. Here, we use AcrIF7 to explore the dissemination and molecular evolution of an anti-CRISPR family. We uncovered 5 subclusters and prevalent anti-CRISPR variants within the group. Remarkably, AcrIF7 homologs display high similarity despite their broad geographical, ecological, and temporal distribution. Although mainly associated with *Pseudomonas aeruginosa*, AcrIF7 was identified in distinct genetic backgrounds indicating horizontal dissemination, primarily by phages. Using mutagenesis, we recreated variation observed in databases but also extended the sequence diversity of the group. Characterisation of the variants identified residues key for the anti-CRISPR function and other contributing to its mutational tolerance. Moreover, molecular docking revealed that variants with affected function lose key interactions with its CRISPR-Cas target. Analysis of publicly available data and the generated variants suggests that the dominant AcrIF7 variant corresponds to the minimal and optimal anti-CRISPR selected in the family. Our study provides a blueprint to investigate the molecular evolution of anti-CRISPR families.

## Abstract 2

Genomics and experimental characterization of the anti-CRISPR gene family AcrIF7 reveal that is mainly mobilized by temperate phages, and is highly conserved due to slow co-evolution with its target. The study also identifies key residues contributing to protein stability and mutational tolerance, and determines the shortest active variant found in genomes.

## Introduction

Bacteria are constantly under attack by bacteriophages. As a result, they have evolved an extensive array of antiphage defence systems such as abortive infection, restriction-modification, and CRISPR-Cas (an acronym for clustered regularly interspaced short palindromic repeats (CRISPR)-associated proteins) [1]. CRISPR-Cas systems are a type of prokaryotic adaptive immune system in which specific sequences targeting foreign nucleic acids are integrated into the bacterial chromosome and provide protection against exogenic mobile elements [2–5]. These systems, present in approximately 40% to 45% of bacterial genomes [6], detect the invader nucleic acid prior to its degradation via an endonuclease. In the opportunistic pathogen *Pseudomonas aeruginosa*, 3 different subtypes of the CRISPR-Cas system have been described to date: I-C, I-E, and I-F, with the latter being present in around 33% of *P*. *aeruginosa* isolates [7,8].

In response to the infection barriers deployed by their hosts, phages have developed various mechanisms to evade defence systems, leading to a dynamic evolutionary arms race [9]. One strategy that phages evolved to circumvent CRISPR-Cas immunity is the use of proteins that block the system, known as anti-CRISPRs (Acr). These proteins, first described in *P*. *aeruginosa* prophages evading the type I-F and I-E systems [10,11], have been identified in mobile genetic elements such as phages and plasmids, but are also encoded in bacterial genomes. In fact, it has been proposed that Acr proteins are present in more than 30% of *P*. *aeruginosa* strains that carry CRISPR-Cas systems [8].

Since their discovery, anti-CRISPR research has mostly centred on identifying new anti-CRISPR genes in diverse bacterial species [12,13]. Over 90 anti-CRISPR families evading different CRISPR-Cas system types have been reported [14] (http://guolab.whu.edu.cn/anti-CRISPRdb/), of which at least 24 belong to the AcrIF family. These families rarely share sequence similarity and seem to possess distinctive molecular mechanisms of action. As the number of reported anti-CRISPRs keeps rising, efforts have been put into compiling and organising anti-CRISPR sequences and their metadata in the form of a resource database [12]. In its first version, anti-CRISPRdb contained 432 entries, including both experimentally validated and computationally predicted anti-CRISPRs. Currently, this database holds information for more than 3,600 anti-CRISPR proteins corresponding to at least 85 Acr subtypes (http://guolab.whu.edu.cn/anti-CRISPRdb/).

In comparison to the comprehensive efforts put into discovering anti-CRISPR families, the mechanisms of action of these powerful molecules have been investigated to a much lesser extent. Most of the Acr mechanisms characterised to date involve blockage of different steps in the CRISPR-Cas restriction process: DNA binding, cleavage, crRNA loading, or formation of the effector complex [15–17]. For example, AcrIF1 binds Cas7f blocking the recognition of the invading DNA [15], whereas AcrIF2 and AcrIF7 interact with Cas8f impeding DNA binding [15,18]. Additionally, a few anti-CRISPR structural studies have described inter-protein interactions occurring with different components of the CRISPR-Cas system. Some of these reports addressed the identification of important residues in the protein, typically by changing polar amino acids to nonpolar ones by site-directed mutagenesis [18,19].

Besides their molecular mechanism, the evolution of anti-CRISPR families has remained largely unexplored so far. Little is known about the distribution and sequence diversity of anti-CRISPRs belonging to the same family. Still, these attributes are key to understanding how anti-CRISPRs of a certain type are acquired, what their host range is, to what extent their sequences have changed, and whether such changes impact the protein function.

Here, we use AcrIF7 as a model to study the molecular evolution of an anti-CRISPR family. We uncovered the AcrIF7 diversity and distribution by analysing homologs identified in bacterial and phage genomes. We report prevalent sequence variants and show that AcrIF7 homologs display high similarity despite their occurrence in diverse genome regions and wide geographical, ecological, and temporal distribution. Using random and site-directed mutagenesis, we generated observed and novel AcrIF7 variants to investigate the impact of sequence variation in the anti-CRISPR function. Our experimental and computational characterisation not only discovered key residues for the anti-CRISPR function but also distinguished regions contributing to the mutational robustness of the protein. Together, our findings suggest that the dominant AcrIF7 variant represents both the optimal and minimal functional unit of the group and reveal features of AcrIF7 that can be used in favour of its development for biotechnology applications. Furthermore, our study serves as a blueprint to investigate the molecular evolution of other anti-CRISPR families.

## Results

### Protein G2 of phage H70 is an active anti-CRISPR of the family AcrIF7

**Fig 1:**
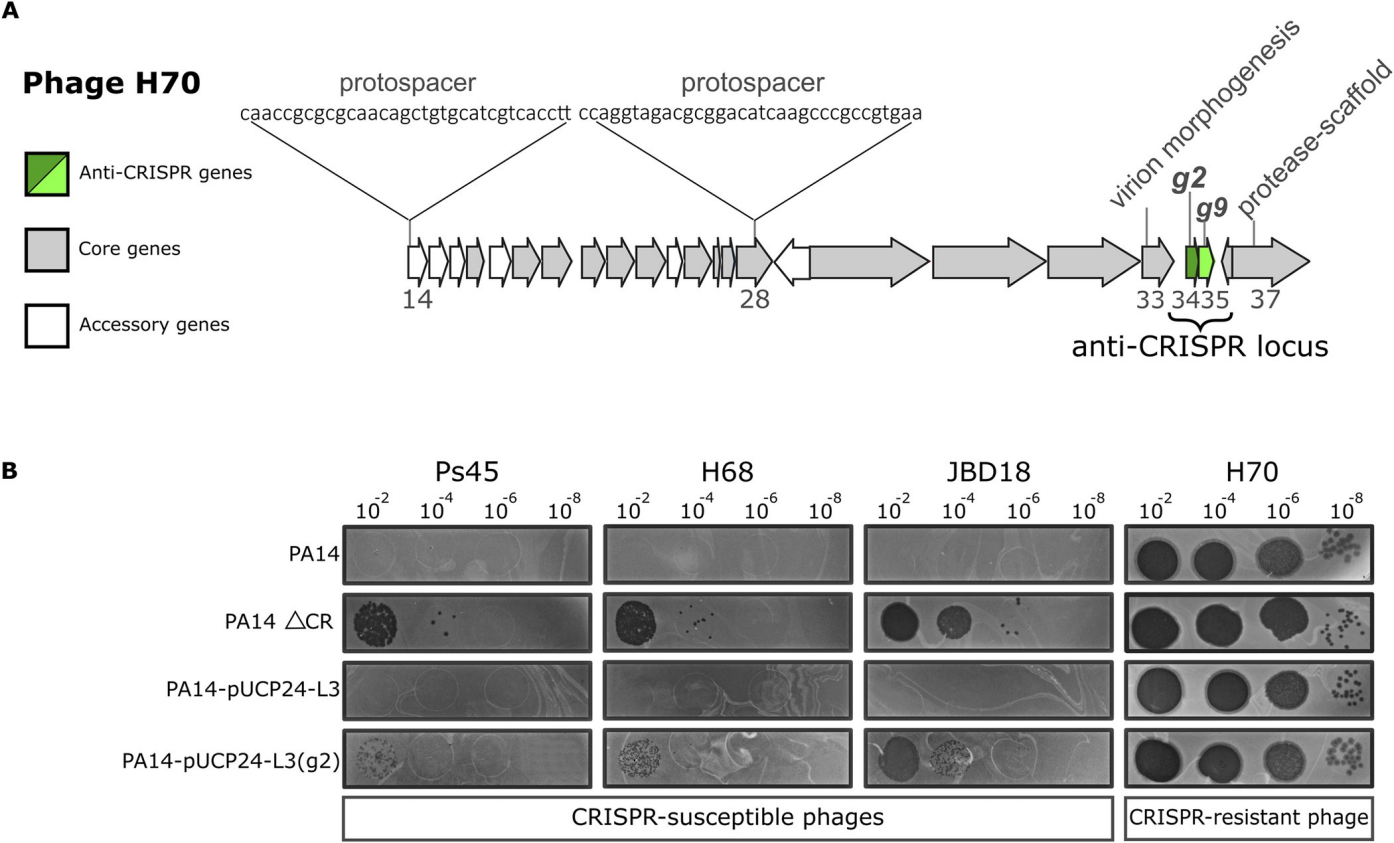
Location of the anti-CRISPR gene *g2* in the genome of phage H70 and inhibition of the CRISPR-Cas system I-F. (A) The map represents a region of the H70 phage genome (*orfs* 14 to 37 shown as arrows). The grey arrows correspond to core genes conserved in the phage group D3112*virus*, whereas the white arrows represent accessory *orfs* [20]. The anti-CRISPR locus, encoding the anti-CRISPR gene (*g2*) and a putative DNA-binding gene (*g9*), are shown in green. (B) Serial dilutions of different CRISPR-sensitive phages (indicated above the figure) were spotted on bacterial lawns of the PA14, PA14 ΔCRISPR-cas (PA14 ΔCR), PA14-pUCP24-L3, and PA14-pUCP24-L3(*g2*) strains. Phage infection (shown as plaques) denotes a lack of CRISPR-Cas defence due to either the absence of the CRISPR-Cas system (PA14 ΔCR) or anti-CRISPR activity (PA14-pUCP24-L3(*g2*)). Note that the titre of each phage stock was different, and therefore, not comparable between phages. CRISPR, clustered regularly interspaced short palindromic repeats.

A genomic analysis of the temperate phage H70 isolated from the *P*. *aeruginosa* clinical strain HIM5 [20] revealed proto-spacers in the *orfs* 14 and 28 matching regions of the CRISPR loci of *P*. *aeruginosa* PA14 (Fig 1A). Yet, infection assays showed that phage H70 could infect the strain PA14 (Fig 1B). Analysis of the phage H70 accessory genome identified an anti-CRISPR locus in the region of genomic plasticity (RGP) G composed of the genes *g2* and *g9* [20], which are homologous to *acrIF7* and *aca1*, respectively [21]. AcrIF7 was first reported as an 83 aa protein (GenBank accession ACD38920.1) with anti-CRISPR activity against the CRISPR-Cas system I-F [12,21]. In comparison, G2 of phage H70 (Accession YP_009152337.1) is 67 aa long, lacking 16 amino acids in the N-terminus of ACD38920.1.

To test the functionality of this shorter version of AcrIF7, we cloned *g2* in the plasmid pUCP24-L3 (S1 Fig) and assessed the protection of CRISPR-sensitive phages against the CRISPR-Cas I-F system of *P*. *aeruginosa*. The infection assays were performed in the strains PA14 wild-type (WT), a PA14 mutant lacking the CRISPR loci and *cas* genes (PA14 ΔCR), PA14 carrying the plasmid with *g2* (PA14-pUCP24-L3(*g2*)), and PA14 transformed with the empty vector (PA14-pUCP24-L3). Phage H70 was able to infect all the strains with similar efficiency, regardless of the presence of the CRISPR-Cas system (Fig 1B). In contrast, CRISPR-sensitive phages JBD18 (that carries a protospacer matching CR1_sp6 [22]), Ps45, and H68 (not sequenced), only produced lytic plaques in the PA14 ΔCR mutant or the strain PA14 WT carrying *g2*; thus demonstrating that this shorter version of AcrIF7 is a functional anti-CRISPR (Fig 1B).

### AcrIF7 family is conserved and mainly associated with *P*. *aeruginosa* prophages

**Fig 2:**
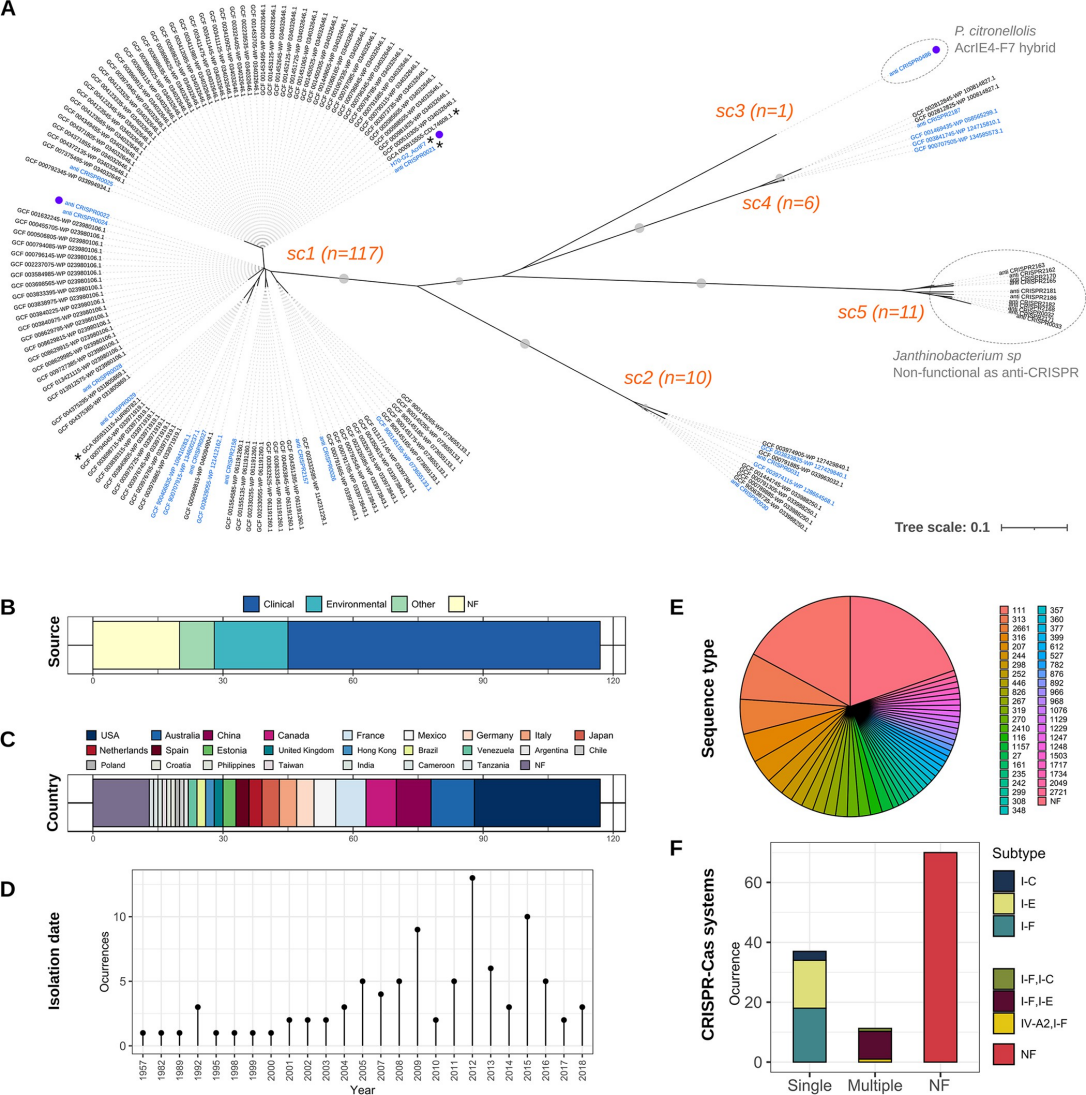
Diversity of members of the anti-CRISPR family AcrIF7. (A) Neighbour-joining unrooted tree displaying the patterns of sequence similarity among protein sequences homologous to G2 of phage H70. Homologous sequences were identified through BLASTp searches against anti-CRISPRdb [12] and proteomes of *Pseudomonas* phages and *P*. *aeruginosa* genomes from GenBank (see Methods). The 145 amino acid sequences presented in the tree were aligned with PRALINE [25]. The tree was inferred from the resulting alignment with Seaview v4.6 [26] (BioNJ method). Grey dots on tree branches represent bootstrap support values >80 calculated from 1,000 replicates. Subclusters (sc) identified in the tree are indicated in orange along with the number of sequences in them. The 25 nonredundant sequences from anti-CRISPRdb are labelled with their corresponding identifier in the database (“anti_CRISPR” prefix). The remaining sequence labels indicate the GenBank assembly identifier and protein accession number separated by a hyphen (“-”) except for the sequence corresponding to G2. Asterisks mark sequences identified in phage genomes, whereas purple dots pinpoint sequences that have been experimentally verified as an anti-CRISPR. Labels in blue denote nonredundant sequences within their corresponding subcluster (excluding those in sc5) and thus represent the diversity of protein sequences in the tree. Dotted line circles indicate sequences identified in non-*P*. *aeruginosa* genomes. Notes on the hybrid nature of the sequence in sc3, and the lack of identifiable anti-CRISPR activity against the systems I-F and I-E of *P*. *aeruginosa* in a homolog (accession: WP_034755374.1) of sc5, correspond to references [27] and [21]. (B–F) Metadata associated with the 117 *P*. *aeruginosa* genomes encoding an AcrIF7 homolog. Data plotted in panels B–D (bacterial strains source, country and year of isolation, respectively) were extracted from the genomes BioSample record (S1 Data). Sequence types (ST) presented in panel E were identified using the pubMLST *P*. *aeruginosa* scheme ([28], http://pubmlst.org/paeruginosa) and the mlst tool v.2.8 ([29], https://github.com/tseemann/mlst). The occurrence of CRISPR-Cas systems in the AcrIF7-carrier genomes, displayed in panel F, was assessed with cctyper v1.4.4 [30]. CRISPR, clustered regularly interspaced short palindromic repeats; MLST, multilocus sequence typing.

Since the major difference between G2 and the first reported AcrIF7 is the additional 16 amino acids in the N-terminus of ACD38920.1, we sought to investigate the diversity of this anti-CRISPR family. A comparative search against the approximately 3,700 sequences available in anti-CRISPRdb [12] only identified proteins of the AcrIF7 family as homologous to G2. The 68 homologs identified in the database, corresponding to 25 unique sequences, included a pair of chimeric proteins with homology to anti-CRISPR of the families IE4 and IF7. Members of the AcrIF7 family reported in anti-CRISPRdb are mostly associated with *P*. *aeruginosa* genomes but also found in different *Janthinobacterium* species and in *P*. *citronellolis*, with the latter corresponding to AcrIE4-F7 anti-CRISPR hybrids (Fig 2A) [23].

To further explore the diversity and distribution of the AcrIF7 family, we expanded our homology search to all proteins encoded in *Pseudomonas* phage genomes deposited in GenBank (*n* = 574) and *P*. *aeruginosa* genomes available in RefSeq (*n* = 5,279). One hundred nineteen homologs were identified, 2 in phages and 117 in *P*. *aeruginosa* genomes (corresponding to 2.2% - 117/5,279), 28 of which also carried a CRISPR-Cas system I-F (Fig 2F). Multilocus sequence typing (MLST) analysis of the bacterial sequences distinguished 44 ST types in 94 genomes, with the remaining 23 sequences missing 1 or 2 alleles, thereby highlighting the diversity of *P*. *aeruginosa* isolates carrying an anti-CRISPR of the AcrIF7 family (Fig 2E; S1 Data). Likewise, analysis of metadata retrieved for the *P*. *aeruginosa* genomes revealed an extensive geographical, temporal, and source distribution encompassing 5 continents, over 6 decades, and a large variety of clinical and environmental samples (Fig 2B–2D; S1 Data).

Comparison of the 119 AcrIF7 homologs identified in phage and *P*. *aeruginosa* genomes with the 25 nonredundant protein sequences from anti-CRISPRdb and that from phage H70 uncovered 5 subclusters within the family (Fig 2A). Three subclusters (sc1, sc2, and sc4) were detected in *P*. *aeruginosa* and *Pseudomonas* phage genomes, with sc1 representing the dominant type. A BLAST search at nucleotide level against non-*P*. *aeruginosa* bacterial and phage genomes in GenBank using a representative from each of the 5 subclusters only identified a match of sc3 in *P*. *citronellolis* (accession: CP015878.1) and confirmed that sc5 is associated with *Janthinobacterium* species. No matches were detected in plasmid sequences reported in pATLAS [24].

**Fig 3:**
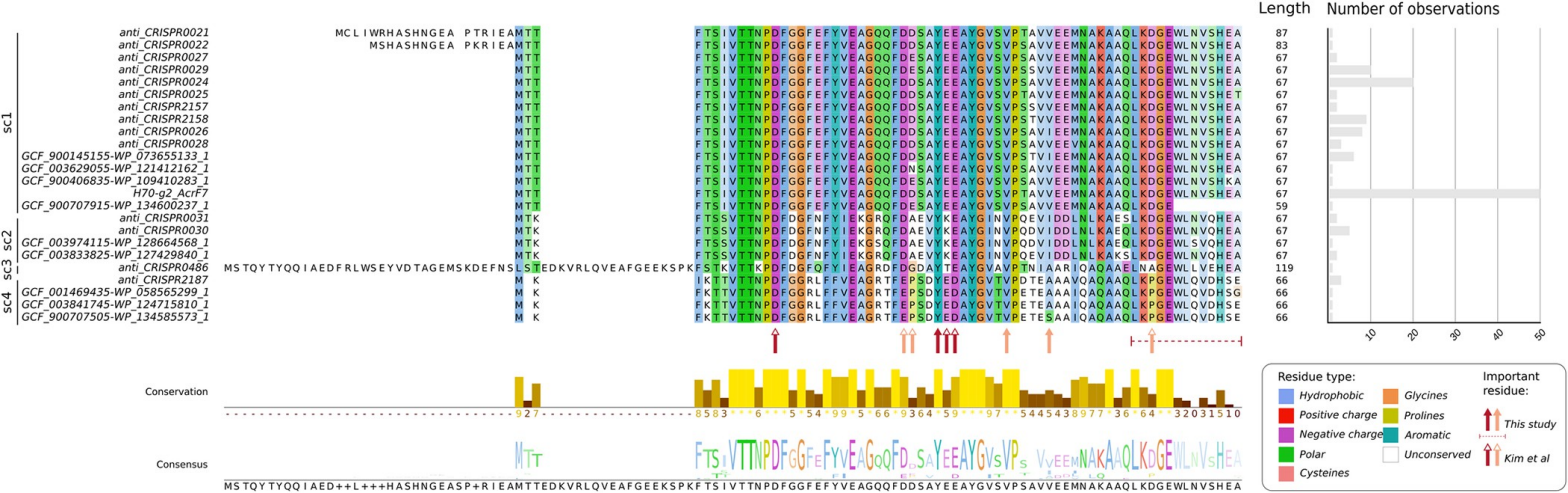
Alignment of nonredundant protein sequences of the AcrIF7 family. The 24 nonredundant protein sequences selected as representative of the diversity observed among AcrIF7 homologs (excluding sc5; see Methods and sequence labels in blue in Fig 2) were aligned with PRALINE [25]. The resulting alignment was visualised with Jalview v2.11.1.4 [31]. Identifiers of the homologous variants, shown on the left side of the alignment, correspond to those described in Fig 2. The subcluster to which the variant belongs is indicated next to its identifier. The length of each variant sequence is displayed on the right side of the alignment, next to the bar plot illustrating the number of observations of the different variants among the genomes where a G2 homolog was identified (see Fig 2, Table B in S2 Data). Residues in the alignment are colour coded based on their level of conservation in a given position and the residue type they belong to according to the ClustalX shading scheme, indicated at the bottom-right of the figure. The conservation level and consensus sequence of the alignment are represented with a bar plot and sequence logo at the bottom of the figure, respectively. Residues identified in this study as important for the G2 anti-CRISPR activity are pinpointed with solid arrows or a dotted line below the alignment. The dotted line indicates that the lack of the underscored residues in G2 nullifies the anti-CRISPR activity of the protein (see Fig 5). Residues important for the AcrIF7-CRISPR-Cas interaction as reported by Kim and colleagues [18] are identified with open arrows. Red arrows denote residues on which mutations drive the loss of the AcrIF7 function or interaction, whereas orange arrows indicate residues on which mutations have a partial effect. CRISPR, clustered regularly interspaced short palindromic repeats.

Members in the subcluster 5 (11 sequences in Fig 2A) were excluded from further analyses because no anti-CRISPR activity against *Pseudomonas* was detected in previous experimental characterisation [21]. Comparison of the remaining 134 protein sequences from subclusters 1 to 4 showed that sequence similarity ranged from 62% to 81% between subclusters (Table A in S2 Data) and uncovered 24 nonredundant sequences representing the diversity of the family (Figs 2 and 3). Notably, AcrIF7 members of the same subcluster displayed limited sequence variation, with similarity values ranging from 98% to 100%, corresponding to 5 to 14 mutations (Fig 3). In the dominant type sc1, containing 117 members and including G2 of phage H70, only 15 different sequences and 14 mutations were distinguished (Fig 3). Sequences within this subcluster varied from 59 to 87 amino acids long, with 67 aa representing the predominant length (Fig 3). Remarkably, the prevalence of variants within the dominant AcrIF7 type sc1 also differed considerably; variants represented by G2 and anti_CRISPR0024, separated by a single mutation, corresponded to 43% and 17% of the members in the subcluster, respectively (Fig 3).

**Fig 4:**
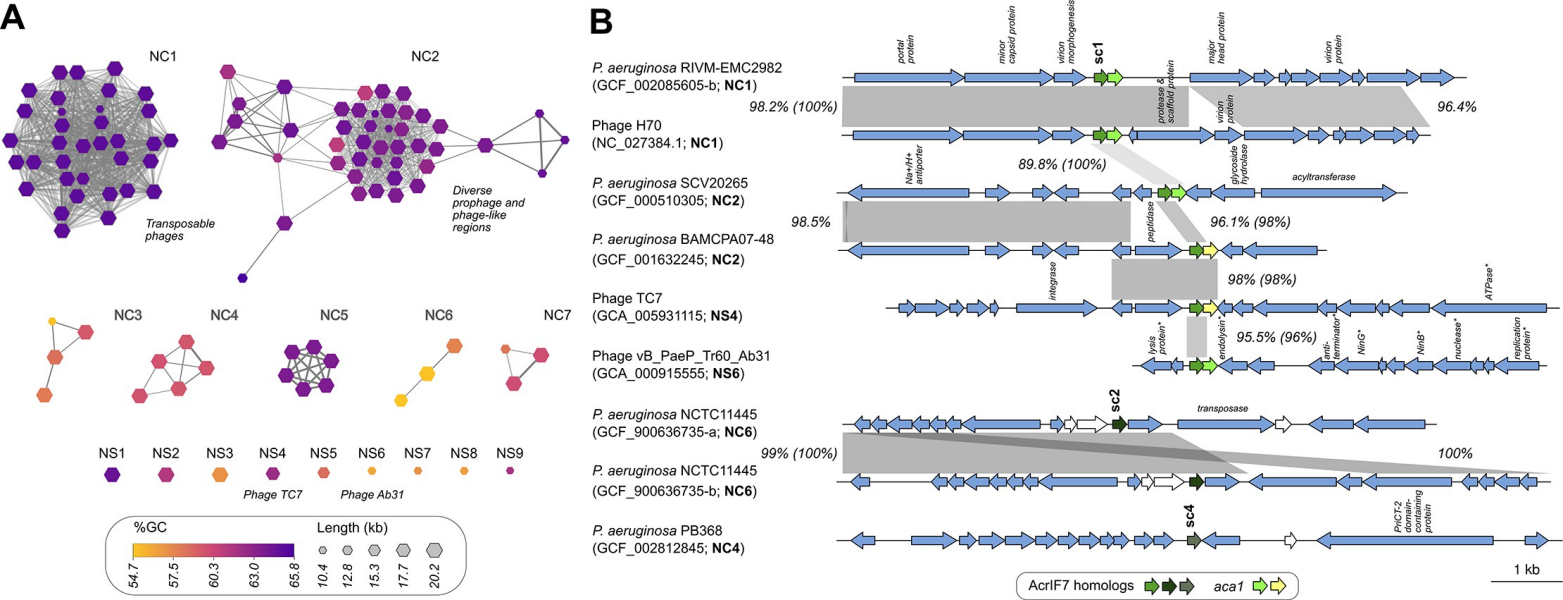
Comparative analysis of genome regions harbouring AcrIF7 homologs. The figure shows a similarity network (A) and pairwise comparisons (B) at the nucleotide level of regions containing an AcrIF7 homolog in *P*. *aeruginosa* and phage genomes. (A) One hundred nine regions containing an AcrIF7 homolog gene plus at least 10 kb of flanking sequence (where available; see Methods) were extracted and compared all-vs-all with mash. Regions were then clustered based on mutation distance and visualised in a network to determine their diversity and frequency among the set of analysed genomes. Connected components clustering identified the clusters (NC) and singletons (NS) in the network. GC content and size of the compared region are indicated below the network. (B) Regions from complete genomes selected for pairwise comparison are paired with their closest match. Only 5 kb of each flanking side of the regions are shown. The organism name, GenBank assembly accession and Network Cluster or Singleton (in parenthesis) of the regions are indicated next to their corresponding gene maps. Instances where more than one AcrIF7 homolog was detected in the same genome are distinguished with a suffix letter added to the GenBank accession number. The AcrIF7 homolog genes and *aca1* are colour coded as indicated in the figure. The subcluster type of the different AcrIF7 homolog genes (see Fig 2) is shown above the corresponding gene arrow. Where available, functions assigned to ORF products, as indicated in the GenBank file, are displayed above the corresponding arrow. Asterisks mark functions assigned as putative. Light yellow arrows denote ORFs encoding homologs of Aca1 overlooked in the original annotations. White arrows indicate overlooked ORFs with unknown functions. The percentage of sequence identity detected between homologous regions, depicted as grey connecting blocks, is indicated next to the corresponding block. For homologous regions containing an AcrIF7 homolog gene, the percentage of identity between the gene sequences is additionally indicated in parenthesis.

We then investigated whether sequence conservation observed among homologs of the same subcluster was extended to the genome regions encoding the anti-CRISPR gene. We extracted and compared regions flanking anti-CRISPRs of the AcrIF7 family identified in phage and *P*. *aeruginosa* genomes. The results of the comparative analysis were used to generate a similarity network representing both the diversity and incidence of genomic backgrounds encoding AcrIF7 (Fig 4A). The network uncovered 7 clusters and 9 singletons, thereby indicating that AcrIF7 is associated with various genomic backgrounds, indicative of anti-CRISPR acquisition via horizontal gene transfer. Importantly, the diversity of genetic contexts in which AcrIF7 occurs is further highlighted by the spectrum of GC content of its flanking regions, ranging from 55% to 66% (Fig 4A).

AcrIF7 was identified in 3 distinct known phage types, 2 of which, represented by the siphophage H70 and podophage Ab31, are reported as temperate phages [18,32]; the third one, TC7, has not been characterised. Still, our network clustering revealed that AcrIF7 is strongly associated with closely related transposable phages of the group D3112*virus*, which form the network cluster 1 (NC1, Fig 4A) and are represented by phage H70; thus implying that this phage group represents the main reservoir and mobile platform for this anti-CRISPR family in *P*. *aeruginosa*. In line with this observation, phages Ab31 and TC7 correspond to singletons in the network (NS4 and NS6).

We also found a second large cluster in the network, NC2 (Fig 4A), formed by regions dissimilar to plasmids or phages reported previously. To gain insights into the nature of the regions, we inspected the annotations of 4 of them. We identified functions indicative of bacterial genes upstream the gene *acrIF7* (Tables A and B in S3 Data) and some phage-related functions downstream. Further inspection of the downstream regions, beyond what was originally extracted for the comparative analysis, unveiled prophage, and phage-like regions of approximately 41 kb in size (see Methods, S2 Fig, and Table B in S3 Data). Comparison of the prophage sequences showed that they bear limited similarity to each other (S2 Fig) or to phages deposited in GenBank (Tables A and B in S3 Data), and therefore, represent novel unrelated phage groups. Together, these results suggest that NC2 is composed of diverse prophages inserted in similar chromosomal positions (Fig 4B). Identification of potential attachment sites in one of the prophage regions indicates that AcrIF7 is encoded in the prophage and hints a similar scenario for the other phage regions in NC2.

Surprisingly, the comparative analysis also uncovered the presence of more than 1 copy of the *acrIF7* gene in the genome of 3 *P*. *aeruginosa* strains: RIVM-EMC2982 (GCF_002085605), Carb01 63 (GCF_000981825), and NCTC11445 (GCF_900636735). In these cases, the anti-CRISPR gene was located in different genome positions, and it was frequently surrounded by homologous phage genes.

In terms of neighbour genes, no other anti-CRISPRs were detected next to the gene *acrIF7* in the regions analysed, but *aca1* was typically located immediately downstream of *acrIF7* genes of the subcluster sc1 (Fig 4B), as it has been reported for the member of sc3 [23]. In contrast, no homologs of Aca1, nor proteins with a lambda repressor-like DNA-binding domain characteristic of Aca1 were identified in the regions flanking members of the subclusters 2, 4, and 5. Despite the existence of sequence variation between the compared regions, the anti-CRISPR gene commonly bore no mutations or displayed nucleotide sequence conservation above the average (Fig 4B). For example, the 204 bp coding region of H70 *g2* was identical in 11 genomes, including in the unrelated regions of *P*. *aeruginosa* SCV20265 prophage and phage Ab31. Notably, the high level of conservation in the *acrIF7* coding sequence did not span its neighbour gene *aca1* in our pairwise comparisons (Fig 4B). To explore the extent of this observation, we identified, compared, and calculated the similarity of Aca1 protein homologs encoded in *P*. *aeruginosa* phage and bacterial genomes (S3 Fig). Our results show that Aca1 is indeed less conserved than AcrIF7; hence, we speculate that these genes are subject to different selective pressures.

### Functional characterisation of AcrIF7 variants reveals amino acids contributing to mutational tolerance and protein stability

**Fig 5:**
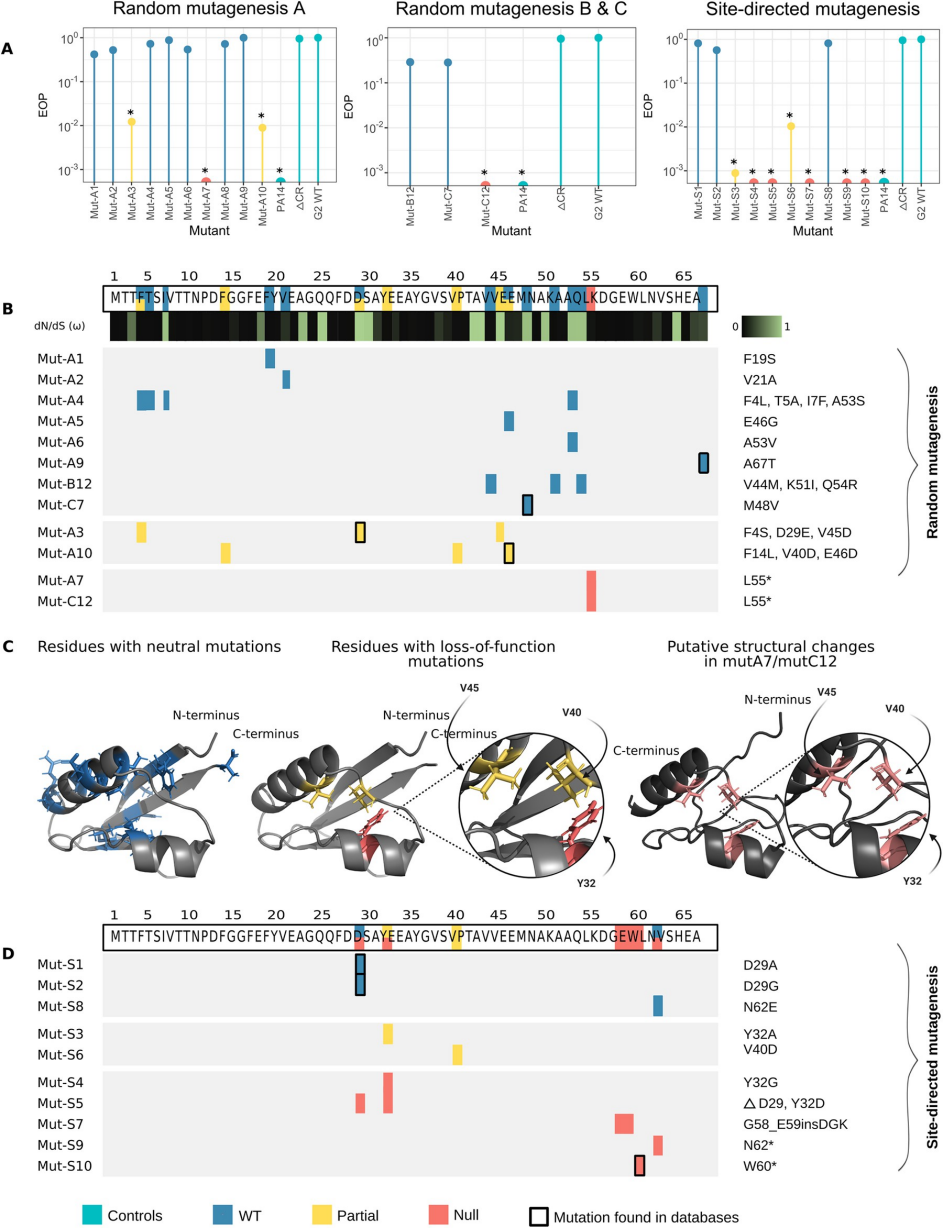
Impact of genetic variation on AcrIF7 function and structure. (A) Efficiency of G2 mutants at inhibiting the CRISPR-Cas system I-F. The lollipop charts show the EOP of the CRISPR-sensitive phage JBD18 on PA14 carrying different variants of G2, normalised to the titre of the same phage in PA14 harbouring G2 WT. Asterisks denote adjusted *p*-values ≤0.05 (raw data of replicates and *p*-values can be found in S5 Data). (B) Mutational map of G2 variants generated by random mutagenesis. The colours represent the phenotype: wild-type (in blue), partial loss-of-function (in yellow), or null activity (in red). The changes in each mutant are shown next to the map (e.g., Mut-A1 has a mutation in F19S, whereas Mut-A3 has mutations in F4S, D29E, and V45D). The WT sequence of G2 is displayed at the top of the panel, with each mutated position coloured according to the phenotype of the mutant that carried changes in that position. Below the sequence, a heatmap is shown representing the dN/dS value for each of the amino acids in G2 (S6 Data). Black indicated a strong negative selection, whereas green symbolises a neutral selection. Rectangles with a bold black contour indicate that those specific mutations (both position and amino acid change) were found in sequences in the databases. (C) AlphaFold2 prediction of G2 structure showing residues with neutral mutations (in blue) or loss-of-function mutations (in yellow or red). Protein model prediction for the mutants mutA/mutC12 lacking 13 amino acids in the C-terminus. Amino acids in pink correspond to loss-of-function mutations; the figure shows the displacement of Y32 in the structure of the mutant, while V45 and V40 remain in the same predicted position as G2 WT. (D) Mutational map of G2 variants generated by site-directed mutagenesis. The figure shows the mutations recreated based on the results of the random mutagenesis and the AlphaFold structures. CRISPR, clustered regularly interspaced short palindromic repeats; EOP, efficiency of plating; WT, wild-type.

Screening of the AcrIF7 sequences for the presence of known protein signatures or classification into established protein families yielded no results. Thus, no functional domains or motifs, nor links to functionally characterised protein sequences were identified. Furthermore, the high levels of sequence similarity observed among AcrIF7 homologs hampered the discrimination of conserved positions in the anti-CRISPR protein that could be important for its function. Since the goal of this study was to investigate how sequence variation affects the anti-CRISPR function, but the diversity was rather limited, we decided to undertake an unbiased approach similar to directed evolution to explore the mutational landscape of G2. We used a random mutagenesis strategy, in which we implemented error-prone conditions during the PCR to promote the misincorporation of nucleotides by the polymerase (32). Three different mutagenic conditions were applied to widen the error rate spectrum (S4 Data), which ranged from 0.6% to 1.4% per position. We then functionally characterised the resulting library, detecting mutations that impaired the protein function and some others with a neutral effect (Figs 5 and S4–S6).

Most of the tested mutant candidates displayed a WT phenotype (i.e., JBD18 was able to infect the strain PA14 WT carrying the anti-CRISPR variant with the same efficiency as the strain containing the WT G2—S5 Data), which harboured mutations scattered across the G2 sequence (Fig 5). Two mutants displayed a partial loss-of-function (Mut-A3 and Mut-A10). Although JBD18 efficiency of plating (EOP) decreased approximately 100-fold in the partially functional mutants, the phage could infect the PA14 strain, indicating a residual protection effect provided by these G2 variants (Fig 5A). Mut-A3 featured 3 point mutations: F4S, D29E, and V45D. The change in residue 29 of aspartate for glutamate is not expected to impact the protein function as both amino acids are chemically similar. As for the mutation F4S, a mutant with WT phenotype (Mut-A4) also featured a mutation in the same position, thus pinpointing the change in the valine 45 for an aspartic acid (V45D) as the most likely driver of the reduction in anti-CRISPR activity observed in Mut-A3 (Fig 5B). Similarly, Mut-A10 carried the mutations F14L, V40D, and E46D, from which the mutation V40D is expected to have a larger impact since a nonpolar amino acid was replaced by an acidic one. Two mutants featured null anti-CRISPR activity: Mut-A7 and Mut-C12 (Fig 5A). Both null mutants acquired mutations that resulted in the introduction of a premature stop codon in position 55 (L55*), albeit in a different manner (Fig 5B). The premature stop codon resulted in the deletion of 13 amino acids of G2, which could potentially alter its tertiary structure (S7 Fig) and, therefore, the interaction with Cas8f.

The relative rates of non-synonymous to synonymous mutations (dN/dS) were determined to estimate the selective pressure of the residues in the AcrIF7 family (Fig 5B-heatmap, S6 Data). We found that our mutagenesis results correlate with dN/dS values obtained from the natural variants: changes in amino acids with a neutral effect on the protein tend to have a dN/dS value of approximately 1, indicative of neutral evolution. Whereas the dN/dS values for amino acids mutated in partial or null mutants were, in general, closer to 0, suggesting a negative selection.

Analysis of the structure model of G2 predicted by AlphaFold2 [33] showed that residues with experimentally identified neutral mutations, i.e., those that did not affect the anti-CRISPR activity, were dispersed in the protein structure (Fig 5C, residues in blue). On the other hand, residues with mutations that we predicted to cause a partial loss of function in Mut-A3 and Mut-A10, namely V40 and V45, were located closely in the structure model (Fig 5C, residues highlighted in red). Intriguingly, V40 and V45 were tightly clustered with a tyrosine located in the short alpha-helix (Y32) in the interior of the protein (Fig 5C). Therefore, we hypothesised this amino acid could also be necessary for the anti-CRISPR function. To test our hypothesis, we created a new series of mutants by site-directed mutagenesis (identified as Mut-S in Fig 5D). We changed Y32 for A and G (Mut-S3 and Mut-S4, respectively) observing a 1,000-fold reduction in the anti-CRISPR activity or no activity at all, respectively (Fig 5D). These results indicate that mutations in Y32 have a negative effect on the protein function and therefore explain why this residue is conserved among the members of the family (Figs 3 and 5B-heatmap).

Next, we assessed whether the mutation V40D drove the partial loss of function previously observed in Mut-A10 that carried 2 additional mutations. The new mutant Mut-S6 (V40D) exhibited a 100-fold reduction in the EOP (Fig 5D). Additionally, since D29 was one of most variable residues in the AcrIF7 family (Figs 3 and 5B-heatmap), we replaced it with A or G (Mut-S1 and Mut-S2, respectively) to test its functional impact. We observed no significant differences in the anti-CRISPR activity of these mutants, thus confirming that mutations in D29 do not influence the function (Fig 5D). Moreover, it suggests that the mutation V45D caused the partial loss of function in Mut-A3. Altogether, the evidence shows that Y32 and V40 are functionally important, and V45 is likely to have a similar contribution as V40 to the protein function. Together, they likely play an important role in stabilising the protein structure.

**Fig 6:**
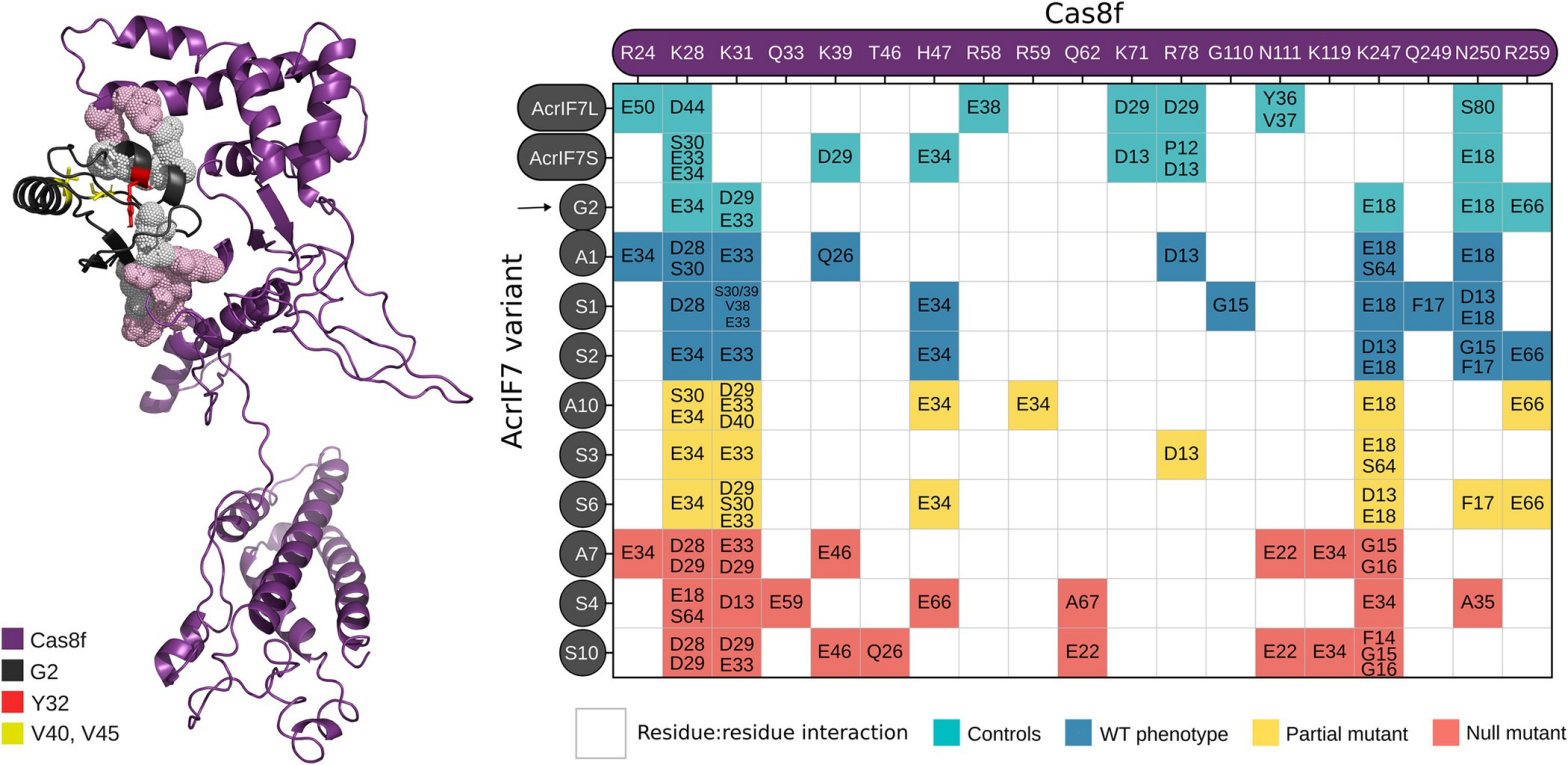
Residue-residue interactions of AcrIF7 variants with the Cascade complex. The interaction of the AlphaFold model of G2 and Cas8f is shown in the left panel. In the right panel, the matrix shows the residue–residue interactions between the AcrIF7 mutants and Cas8f. At the top, the residues of Cas8f that interact with AcrIF7 are displayed, whereas the panel on the left represents each of the AcrIF7 variants assessed in the docking analysis. The numbers inside the squared denote the interacting AcrIF7 residue(s); e.g., residue R24 of Cas8f interacts with the residue E50 from the long AcrIF7 (AcrIF7L), and E34 from MutA1 and MutA7, whereas it does not interact with any residues of G2 WT. The colour of the squares reflects the anti-CRISPR activity of the variant: blue for wild-type, yellow for partial loss of function, and red for null mutants. The proteins used for the analysis were AcrIF7L (7JZX) [36], AcrIF7S (6M3N) [18], and G2 and with all the variants we generated (AlphaFold model). CRISPR, clustered regularly interspaced short palindromic repeats.

To understand the molecular basis of the loss of anti-CRISPR function, we performed docking analyses of the mutants in interaction with Cas8f (Fig 6). Our analysis of the mutants in Y32 (Mut-S3 and Mut-S4) predicted that they lost the interaction with R259 in Cas8f, which is part of the positively charged channel responsible for the non-target DNA displacement [34] (Fig 6). Similarly, the residue–residue interaction analyses revealed that that both Mut-S6 and Mut-A10 carrying a mutation in V40 lost the AcrIF7:E18-Cas8f:N250 interaction. This is consistent with the observations of Guo and colleagues [35] who found that Cas8f N111 and N250 are responsible for the recognition of the PAM duplex on target DNA and essential for CRISPR activity. Our analysis of the functionally active mutants demonstrated that the interactions with K28, K31, N250, and R259 of Cas8f are conserved. This suggests that the negatively charged surface of AcrIF7 is important for binding the positively charged channel of Cas8f and thus for anti-CRISPR activity, consistent with Kim and colleagues and Gabel and colleagues findings (Fig 6) [18,36].

We further investigated the importance of protein length since this feature was variable among the members of the AcrIF7 family (Fig 3), and our null mutants Mut-A7/C12 were shorter in the carboxy-terminus (Fig 5). We introduced stop codons in positions 60 and 62 and tested whether the variants were still functional. We found that mutants Mut-S9 (identical to the natural variant GCF_900707915-WP_134600237_1) and Mut-S10 that lost 8 and 6 amino acids, respectively, were inactive (Fig 5). Interestingly, changing the residue in position 62 (N62E, mutant Mut-S8) had a neutral effect on the anti-CRISPR activity, implicating that loss of residues in the carboxyl terminus and the subsequent loss of a beta strand which could destabilise the structure (Figs 5 and S7), is the main factor driving the protein inactivation. Together with the analysis of the diversity of the AcrIF7 family, these results suggest that 67 amino acids constitute the minimal functional AcrIF7 found in nature, although this may not be the absolute minimal version of AcrIF7.

In summary, we assessed the impact of sequence variation on the function of AcrIF7. We introduced 30 different mutations in 21 positions scattered throughout the anti-CRISPR gene (Fig 5), corresponding to 31.3% of the protein. Seven of the mutations generated in vitro were naturally present in the AcrIF7 family (Figs 5B and 3), with 6 of them displaying a WT phenotype. Notably, mutations introduced in 14 different positions, corresponding to 66.6% of the mutated residues, showed a neutral effect on the protein function, suggesting that these positions contribute to the mutational tolerance of AcrIF7. Additionally, by using docking analysis, we predicted how sequence divergence affects the interaction with Cas8f.

### Absence of a CRISPR-Cas system does not promote rapid accumulation of mutations in the AcrIF7 anti-CRISPR locus

Given that the AcrIF7 seemed highly conserved despite the diversity of the strains (ST, isolation source, geographic location, and temporality) and backgrounds where it was found (different types of phages), we explored the molecular mechanisms that could drive such conservation. First, we investigated whether the natural diversity of AcrIF7 was due to the natural diversity of Cas8f. We identified 1,911 Cas8f homologs in *P*. *aeruginosa* genomes. However, we only detected 28 instances of a genome encoding both Cas8f and AcrIF7 (Figs 2F and S3). This result is in line with our search of CRISPR-Cas systems showing that more than half of the genomes where we found the anti-CRISPR do not carry an identifiable complete CRISPR-Cas system (Fig 2F). It is worth noting that in isolates with Cas8f, only AcrIF7 of the subclusters sc1 and sc2 were identified (S1 Data). Interestingly, our comparison of Cas8f homologs revealed a high level of conservation in both sequence and length, even higher than for AcrIF7 (S3 Fig).

**Fig 7:**
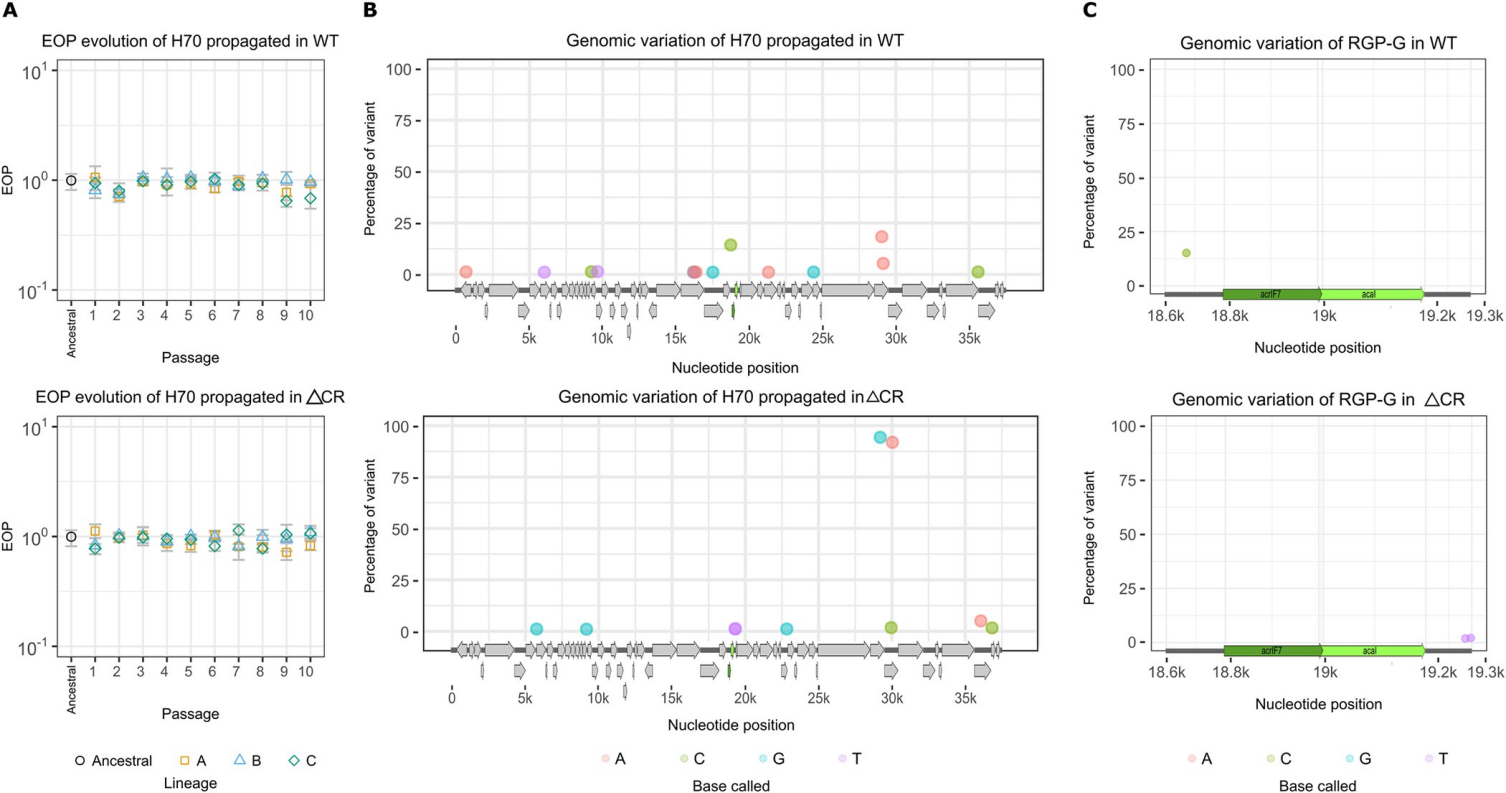
Experimental evolution of H70 in PA14 WT and PA14 ΔCR. Panel (A) illustrates the evolution of the EOP throughout the passages in either PA14 WT (top) or PA14 ΔCR (bottom). Each coloured shape represents a different lineage (biological replicates) with 3 technical replicates each. No statistically significant difference in the EOP was found in one-way ANOVA tests. Panel (B) shows the unique variants (only present in one of the populations) in the H70 genome (from the last passage) that differ from the reference (NC_027384.1) and comprise more than 1% of the population. Panel (C) represents the variants found in the RGP G that is composed of the genes *acrIF7* (*g2*) and *aca1* (*g9*), and their respective intergenic regions. EOP, efficiency of plating; RGP, region of genomic plasticity; WT, wild-type.

Therefore, we hypothesised that the conservation of Cas8f may be an important factor in the observed AcrIF7 diversity. To test this, we investigated whether the absence of the target would promote anti-CRISPR variation in an evolution experiment. If the reason why the anti-CRISPR was so conserved was the presence of the CRISPR-Cas system, in its absence, we would observe sequence divergence of AcrIF7, product of arising mutations not being selected against. We serially propagated the phage H70 (that carries *g2*) in either PA14 WT or PA14 ΔCR and determined the efficiency of the evolved phage lineages to evade the CRISPR-Cas system (Fig 7A, Table A in S7 Data). No statistically significant differences in the EOP were found among any of the evolved stocks (*p*-value of 0.6468 for the phage evolved in PA14 WT and 0.7088 for the one propagated on PA14 ΔCR), indicating that the efficiency of AcrIF7 to inhibit the CRISPR-Cas system remained stable throughout the passages regardless of the absence of an active CRISPR-Cas system (Fig 7A).

Deep whole-genome sequencing and variant calling identified 14 unique mutations in the phage propagated on PA14 WT and 10 in the phage evolved on PA14 ΔCR (Fig 7B, Tables B and C in S7 Data). Additionally, we identified 64 were shared between the 2 populations (Table D in S7 Data). The most variable gene in the population replicated in PA14 WT was the *orf* 31, encoding the portal protein, carrying 3 variants (Table B in S7 Data). On the other hand, in PA14 ΔCR, the *orf* 50, encoding a hypothetical protein, was the most variable gene with 2 mutations. By contrast, *g2* did not display any mutations in either of the populations (Fig 7C). In the rest of the RGP-G, which comprises the genes *acrIF7* and *aca1*, we found 1 mutation in the population passaged on PA14 WT in the upstream region of *acrIF7* and 2 in the phage stock coming from PA14 ΔCR in the downstream *aca1* (Fig 7C and Tables B and C in S7 Data). These mutations are not likely to impact the anti-CRISPR activity as they were not located in the coding region or nearby the promoter or RBS, thus correlating with the phenotypes from the EOP experiments. These results show that in the absence of the CRISPR-Cas system, and Cas8f in specific, AcrIF7 remains conserved for at least around 120 generations.

Altogether, our results show that members of the AcrIF7 family are highly conserved and mobilised primarily by temperate phages. Such conservation is likely the result of the slow coevolution of this family and its CRISPR-Cas target, Cas8f, which shows even higher sequence similarity. Our functional characterisation of AcrIF7 variants suggests that 67 aa is the shortest active variant found in genomes in databases and identified key residues for anti-CRISPR function and mutational tolerance.

## Discussion

CRISPR-Cas systems are widespread in both archaea and bacteria [6], leading to the expectation that a wide variety of anti-CRISPRs exist in phage or other mobile elements to aid them in circumventing this defence system. Consistent with this notion, numerous anti-CRISPRs have been identified recently [12], and it can only be expected that the number of anti-CRISPRs will keep rising rapidly. Still, many questions remain unanswered regarding how these remarkable proteins work, evolve, and spread. In this study, we used AcrIF7 as a model to investigate diversity, distribution, evolution, and functionality within an anti-CRISPR family.

By comparing homologs of the anti-CRISPR *g2* identified in different databases, we portrayed a detailed picture of the diversity within the AcrIF7 family. This enabled the identification of different subclusters and characterisation of their levels of sequence similarity, as well as distinguishing prevalent types and variants representing the diversity of the group (Figs 2A and 3). Our findings indicate that AcrIF7 homologs are mainly associated with *P*. *aeruginosa*, possibly suggesting specialisation to the CRISPR-Cas system of the species in which this anti-CRISPR family was first reported [21]. One exception is the member of the subcluster 3, a hybrid of the anti-CRISPR families IE4 and IF7 found in *P*. *citronellolis*, indicative of the potential flexibility of these molecules [23,27].

Further exploration of regions flanking AcrIF7 homologs in bacterial and phage genomes uncovered that this anti-CRISPR type can be linked to diverse genetic backgrounds in *P*. *aeruginosa* (Fig 4), indicating broad gene mobilisation. For example, we identified AcrIF7 homologs of the subcluster 1 in 3 unrelated phages sharing sequence similarity in less than 2% of their genomes: the siphophage H70 isolated from a clinical strain of *P*. *aeruginosa* in Mexico [20], the podophage Ab31 isolated from wastewater in Ivory Coast [32], and the siphophage TC7 isolated from hospital sewage in China (see metadata in GenBank record MG707188.1). The isolation source of these phages and the *P*. *aeruginosa* isolates carrying AcrIF7 (Fig 2B–2F) outlines the importance of anti-CRISPRs in different environments.

Although AcrIF7 homologs of the subcluster 1 were identified in distinct phages, they were predominantly associated with transposable phages of the type D3112*virus*, of which phage H70 is a member (Fig 4). The genomes of these phages are known to contain multiple regions of genomic plasticity accommodating various accessory genes, with 1 region in particular harbouring diverse arrays of anti-CRISPR genes [10,20]. In fact, anti-CRISPRs were first discovered in D3112*virus* phages [10]. It hence appears that this type of transposable phages represents a major reservoir of anti-CRISPR genes in *P*. *aeruginosa*, which could then be transferred to other mobile elements, consistent with our observation on the abundance of AcrIF7 homologs in D3112*virus* phages compared to other phage types.

Our comparative and network analysis of AcrIF7 flanking regions also uncovered a large cluster (NC2) formed by regions of *P*. *aeruginosa* genomes sharing little or no similarity with previously reported phage or plasmid sequences. Inspection of some regions in NC2 revealed shared bacterial genes upstream *acrIF7* and novel unrelated prophages located downstream the anti-CRISPR gene; thus exposing that NC2 comprises diverse mobile elements inserted in the same relative chromosomal position. Further analysis of regions in NC2 and other clusters and singletons in the network is required to expose the extent of the diversity of elements linked with AcrIF7; however, our results show a remarkable association with phages; hence, implying that the evolution and distribution of AcrIF7 are heavily influenced by the dynamics and range of infection of these viral groups. Unlike other anti-CRISPRs [13,37,38], we did not detect AcrIF7 homologs in virulent phages nor in plasmids. While this observation implies a preferred association with temperate phages, it could also be explained by a limited number of records existing for other types of mobile elements in *P*. *aeruginosa*, especially for plasmids.

The most divergent sequences in the group AcrIF7 are closely related proteins encoded in *Janthinobacterium* sp. genomes, here clustered within the subcluster 5 (sc5; Fig 2). These sequences, however, are nearly identical to a protein that did not exhibit anti-CRISPR activity when tested against the CRISPR-Cas systems I-F and I-E of *P*. *aeruginosa* and I-F of *P*. *atrosepticum* [21]. This suggests that members of the subcluster 5 may either be active against CRISPR-Cas systems of other species or feature an unrelated function. The fact that proteins in sc5 share 62% similarity with those of the subcluster 1 (Table A in S2 Data), and can therefore be easily detected as potential homologs through BLAST searches, prompts us to be cautious about how we infer anti-CRISPR functions from sequence homology information. Since only representatives of the subclusters 1 and 3 in the group AcrIF7 have been experimentally verified as anti-CRISPRs ([14], http://guolab.whu.edu.cn/anti-CRISPRdb/), it remains to be seen whether proteins in the subclusters 2 and 4 share the same function. In line with this remark, no Aca1 homologs were identified adjacent to representatives of sc2 and sc4 analysed here, hinting at either lack of anti-CRISPR activity for proteins in these subclusters or the presence of alternative Aca within the AcrIF7 family.

Random mutagenesis coupled with a selection method is a powerful approach to characterise the fitness landscape of proteins for which limited information is available [39]. This strategy is particularly effective at identifying not only residues involved in protein binding, but also in protein folding, or at finding mutations that enhance protein activity [40–42]. This is relevant for protein families such as AcrIF7, which are highly conserved according to the sequences available in databases, making it difficult to draw inferences about the impact that certain residues have on the protein. However, this approach has also some limitations. For example, it has been previously reported that some anti-CRISPR proteins, such as AcrIIA3 encoded in the genomes of *Listeria* and *Streptococcus* siphophages, are toxic in *E*. *coli* [43], and therefore variants causing toxicity would be negatively selected during the first steps of the process. Nevertheless, by implementing this approach, we not only captured and experimentally characterised some of the mutations naturally observed in AcrIF7 (i.e., there were 2 cases where a natural variant was identical to a mutant obtained in the experiments: anti_CRISPR0027 is identical to Mut-S2, anti_CRISPR0025 is identical to Mut-A9, and other 4 where individual mutations were found), but also generated more variation to study the functionality of a conserved anti-CRISPR. Remarkably, our mutational screening revealed important residues (Y32, V40) contributing to the anti-CRISPR functionality that could not be identified with traditional methods which are focused on testing polar amino acids [18,19]. Additionally, this approach enabled the identification of regions that contribute to the mutational robustness of the protein (Fig 5C, residues in blue).

Essential proteins are typically conserved [44]. This principle may help to understand why some anti-CRISPR families feature high levels of sequence similarity. Although not all phages encode an anti-CRISPR, this function becomes essential when infecting a host with a CRISPR-Cas system. In this context, mutational robustness represents an advantageous trait that minimises the effect of random changes on the protein function [45,46]. Despite the high levels of similarity observed in most of the members of the AcrIF7 family, our experiments identified some mutations with a neutral effect on the anti-CRISPR function. These residues contribute to the mutational tolerance of the protein, corresponding to approximately 67% of the amino acids mutated in our study. Our findings are reminiscent of those reported for the Influenza A virus matrix protein M1, and the middle domain of the heat shock yeast protein Hsp90 [47,48], for which high levels of intrinsic tolerance to mutations were found despite the little sequence variation observed in nature. These proteins play crucial roles in the survival of the microorganism carrying them: AcrIF7 is essential for a successful phage infection in the presence of a CRISPR-Cas system; M1 participates in multiple stages of the viral infectious cycle; Hsp90 is involved in protecting cells from environmental stress and growth at high temperature [47,48]. Future studies focused on deeper mutational scanning of anti-CRISPRs will tell us how robust they are in comparison with other proteins.

Although G2 was capable of carrying multiple mutations without having its function significantly affected (Fig 5, S5 Data), it is possible that those variants are not as stable as the WT version and are therefore negatively selected; thus suggesting that the G2 sequence represents the optimal AcrIF7 version. This notion is supported by the fact that G2 can completely block the CRISPR-Cas system I-F, i.e., a CRISPR-sensitive phage can infect a strain carrying G2 WT with the same efficiency as the mutant lacking the CRISPR-Cas system (Fig 5). Moreover, variants sharing the G2 sequence are the most prevalent among the members of the family (Fig 3), possibly suggesting an evolutionary selection for this version of AcrIF7.

We discovered that Y32 is key for the anti-CRISPR activity of G2. Based on its location inside the short alpha-helix, and our residue–residue interaction analysis predicting no hydrogen bonds, we hypothesise that this residue contributes to the anti-CRISPR activity by stabilising the protein. Likewise, we uncovered that mutations in V40 had a significant impact on the function, likely because of its contribution to the protein hydrophobic packing [49]. This highlights the importance of mutational screening strategies to study anti-CRISPR proteins, as it allowed us to find amino acids that are not part of the protein surface but still contribute largely to its function. Our work also underscores the importance of functional analyses to complement structural studies, as we identified mutations in residues reported to interact with the CRISPR-Cas system (V21, D29) [18,36] that had a neutral effect on the anti-CRISPR function.

We observed that deletions in the Carboxy-terminus of G2 completely abolished the anti-CRISPR function. The structure model of the mutants with the deletion L55* (Mut-A7/C12) suggests that this region is important for the maintenance of the structure and the stability of the protein (S7 Fig). We did not identify longer AcrIF7 variants on the Carboxy-terminus side in databases. In contrast, we found that the anti-CRISPR encoded in the genome GCF_900707915 is shorter. Our characterisation of the mutant Mut-S9 (N62*), however, suggests that this variant is no longer active. Altogether, our mutants characterisation and bioinformatics analyses indicating that the predominant variant length is 67 amino acids (93.6% of the sc1 and nearly 65% of all subclusters), suggest that this protein size has been evolutionarily selected.

It has been proposed that the types of interaction between species determine how fast they coevolve, which can be applied to the interaction between proteins too. We found that Cas8f is even more conserved than AcrIF7, providing a possible explanation for this anti-CRISPR evolution. It is possible that Cas8f is not under selective pressure because there is a plethora of other antiphage systems that provide the cells with protection against mobile genetic elements. Another interesting hypothesis is the Red King effect, which states that organisms (or proteins) with a mutualistic relationship evolve slowly. If this is the case, it would be fascinating to explore how bacteria can potentially benefit from the presence of anti-CRISPRs.

Besides furthering our understanding of the phage-bacteria arms race and coevolution, the study of anti-CRISPRs can boost their application in biotechnology, similar to the recent boom in the development of CRISPR-Cas-based technologies [50]. For example, it has been proposed that anti-CRISPRs can aid the use of CRISPR-Cas9 systems to control gene expression as regulatory tools preventing off-target editing [51]. Additionally, we envisage that anti-CRISPRs can be powerful tools in the fight to control multidrug-resistant bacteria by providing phages engineered for therapy purposes with a gene repertoire enabling them to evade the CRISPR-Cas defence of the target pathogen and increasing their host range. To this end, it is necessary to not only continue the discovery of new anti-CRISPRs but also to thoroughly characterise them and identify their optimal versions for biotechnological use. We focused on the AcrIF7 family because it can efficiently block the most widespread CRISPR-Cas system in *P*. *aeruginosa*. But we consider that a strategy like the one presented in this study, i.e., combining genomics and phylogenomics analyses, mutational scanning, and functional characterisation, can be expanded to other families and be used as a first step towards their biotechnological exploitation.

## Methods

### Bacterial strains, phages, and culture conditions

PA14 WT strain, a mutant lacking the CRISPR-Cas system (PA14 ΔCRISPR loci Δcas genes, also referred to as PA14ΔCR), and the phage JBD18 were kindly provided by Professor Alan R. Davidson [22]. Phage H70 harbouring *acrIF7* gene (named *g2* in the annotation of the phage genome) was isolated from a clinical *P*. *aeruginosa* strain [20] and belonged to Dr. Gabriel Guarneros’ phage collection, along with phages Ps45 and H68. Overnight cultures were grown routinely in LB (Lennox) broth with shaking at 37°C unless otherwise indicated.

### Phage propagation and purification

Phages H70 (G2 carrier) and JBD18 (CRISPR-sensitive) were propagated and purified following the protocol previously reported by Cazares and colleagues [20]. Briefly, the phages were propagated using the standard soft agar overlay method, followed by concentration with PEG and purification by CsCl gradient centrifugation.

### Analysis of AcrIF7, Aca1, and Cas8f sequences

The amino acid sequence of the anti-CRISPR G2 from phage H70 (accession: YP_009152337.1) was first compared against the 3,691 sequences available in anti-CRISPRdb [12] in August 2020 using BLASTp [52]. Only proteins of the AcrIF7 family matched G2. The 68 sequences of proteins of the AcrIF7 family available in the database were clustered using CD-HIT with an identity threshold of 100%, word size of 5, and length difference cutoff of 0 to identify nonredundant sequences (i.e., remove identical sequences). The search of sequences homologous to G2 was extended to 50,457 proteins encoded by 574 *Pseudomonas* phage genomes available in GenBank and 32,262,482 proteins from 5,279 *P*. *aeruginosa* genomes deposited in the GenBank RefSeq database (328 genomes from the complete/chromosome and 4,951 from the scaffold/contig assembly categories) in August 2020. A maximum e-value of 1e-03 was considered to identify homologs in all the BLASTp searches.

A total of 145 amino acid sequences: G2, the 119 homologs detected in *Pseudomonas* phages (*n* = 2) and *P*. *aeruginosa* (*n* = 117) genomes, and the 25 nonredundant AcrIF7 sequences identified in anti-CRISPRdb, were aligned using the PRALINE algorithm [25] with default settings. A neighbour-joining tree was inferred from the multiple sequence alignment using the BioNJ method integrated in Seaview v4.6 [26] with 1,000 bootstrap replicates, observed distance, and including gap sites. The resulting tree was visualised with iTOL v5.7 [53]. One hundred thirty-four homologs from sc1 to sc4 were deduplicated with CD-HIT and the resulting 24 nonredundant sequences aligned with PRALINE as described above; the 11 sequences belonging to the sc5 were excluded from further analyses because 1 representative has been previously described as nonfunctional as anti-CRISPR [21]. The alignment of 24 nonredundant sequences was visualised and edited with Jalview v2.11.1.3 [31]. Protein sequences representative of each subcluster identified in the neighbour-joining tree were scanned against all member databases in InterPro using InterProScan v5.50–84.0 [54] with default settings. The search for homologs in non-*P*. *aeruginosa* genomes was performed at the nucleotide level in the BLASTn suite online [52] with default search parameters, excluding *P*. *aeruginosa* (taxid:287) in the organisms list, and using a representative of each AcrIF7 subcluster as query. AcrIF7 homologs in plasmids were searched with BLASTn against the pATLAS database [24].

Sequences homologous to G9 of phage H70 (Aca1; YP_009152338.1) or Cas8f from *P*. *aeruginosa* PA14 (WP_003139224.1) were searched with BLASTp (maximum e-value of 1e-03) against the collection of phage and bacterial proteomes used to identify AcrIF7 homologs. The sets of identified Aca1 (*n* = 1,507) and Cas8f (*n* = 1,911) homologs, as well as a subset of 133 AcrIF7 homologs encoded in *P*. *aeruginosa* from the complete set of 145 sequences, were aligned using the MUSCLE algorithm [55] with default settings. A neighbour-joining tree of each of the 3 alignments was inferred using the BioNJ method integrated in Seaview v4.6 [26] with 100 bootstrap replicates, observed distance, and including gap sites. The resulting trees were visualised with iTOL v5.7 [53]. Redundant sequences were removed from the alignments in Jalview v2.11.1.3 [31] for both visualisation and to calculate the sequences average conservation: the sum of the conservation scores per column in the alignment divided by the total number of columns (Table D in S8 Data). The alignment conservation score is calculated in Jalview based on the AMAS method [56] and represents the conservation of physico-chemical properties in each column of the alignment.

Nucleotide sequences of the genes encoding the 127 G2 homologs from the subclusters 1 and 2 were extracted from the corresponding genomic fasta files. This dataset includes 4 extra copies of the gene *acrIF7* identified in 3 *P*. *aeruginosa* genomes (GCF_000981825: 2 extra copies, GCF_900636735: 1, GCF_002085605: 1), thus totaling 131 sequences. The sequences were aligned at the protein level with Seaview v4.6 [26] using the PRALINE alignment as a template and the resulting multiple sequence alignment at the nucleotide level was used in the positive selection analysis (see below).

### Comparative analysis of *acrIF7* flanking regions

A sequence stretch including the *acrIF7* CDS region plus 10 kb of the upstream and downstream sequence (i.e., approximately 20 kb in total, when available) was extracted from *P*. *aeruginosa* genomes (*n* = 117) and *Pseudomonas* phage genomes (*n* = 3) identified as carrying an AcrIF7 homolog. Extracted sequences shorter than 10 kb (*n* = 15) were removed. This resulted in 109 regions (Table A in S3 Data), as 3 of the *P*. *aeruginosa* genomes contained extra copies of the gene *acrIF7* (GCF_000981825: 2 extra copies, GCF_900636735: 1, GCF_002085605: 1).

The 109 regions were first compared with mash v2.3 [57]. K-mer-based sequence sketches (s  =  1,000, k  =  21) were generated with the mash sketch algorithm and pairwise mutation distances between sketches were estimated with mash dist using a distance threshold of 0.05 and otherwise default settings. The all-pairs distance matrix obtained was then used for graph visualisation in Cytoscape v3.8.2 [58]. Clusters in the network were defined as connected component clusters. Selected regions were also compared with BLASTn [52] and the pairwise comparisons visualised with the genoPlotR package v0.8.11 [59].

To search for prophages in regions comprising the network cluster 2 (NC2), we first inspected the functions of genes flanking *acrIF7* from the native annotations of 4 regions, namely: GCF_000510305-NC_023149.1, GCF_001632245-NZ_CP015377.1, GCF_003632525-NZ_QZXW01000315.1, and GCF_003975725-NZ_RXEF01000001.1. Products related to translation and metabolism were identified upstream *acrIF7* in 3 of the regions (see Table B in S3 Data) and functions related to transmembrane transporters in the other region (GCF_003632525-NZ_QZXW01000315.1). Phage-related functions were detected downstream *acrIF7* in all regions. We then continued searching for phage gene functions downstream *acrIF7*, beyond the 10 kb extracted for the comparative analysis, and stopped when finding functions related to molecular translocation or when reaching the end of the sequence (GCF_003632525-NZ_QZXW01000315.1). The screening resulted in the detection of 4 approximately 41 kb regions featuring phage functions and encompassing *acrIF7*, which were then submitted to PHASTER [60] for prophage prediction (Table B in S3 Data). PHASTER identified prophages in all regions. The completeness score classified 2 of them as intact and the other 2 as questionable. The 4 regions were additionally compared against each other using BLASTn.

To assess whether *acrIF7* was part of phage regions identified in NC2, we delimited the prophage sequence of 1 region. Forty-five kilobases flanking each side of *acrIF7* in GCF_000510305-NC_023149.1 were extracted and compared against the genome of *P*. *aeruginosa* PAO1 (NC_002516.2), which only carries the filamentous prophage Pf4. Homologous regions detected between PAO1 and the extracted region were separated by 42.5 kb, which we predicted correspond to the prophage region. One kilobase of the homologous regions next to each side of the prophage were then compared against each other, leading to the identification of 41 bp nearly identical repeats flanking the prophage and likely corresponding to the attachment sites *att*L and *att*R. The prophage is located at 1,416,053:1,458,658 (including *att*L) and contains an integrase, *acrIF7* and *aca1*.

### Metadata, multilocus sequence typing (MLST), and CRISPR-Cas identification

The MLST profiles of 117 *P*. *aeruginosa* bacterial genomes carrying AcrIF7 were identified from the pubMLST *P*. *aeruginosa* scheme (http://pubmlst.org/paeruginosa/) [61] using the mlst tool v.2.8 (https://github.com/tseemann/mlst) (S1 Data). BioSample records of the genomes were retrieved from GenBank using the NCBI’s Edirect [62] to extract information on the isolation source, year, and country of isolation of the bacterial strains (S1 Data). The genome sequences were also analysed with cctyper v1.4.4 [30] for the identification and subtyping of CRISPR-Cas genes and arrays.

### Random mutagenesis

Error-prone PCR [40] was used to introduce mutations in the sequence. Three different conditions were used (S4 Data), which differ in the concentration of MgCl2, MnCl2, and the number of extension cycles. PCR products were run in a 1% agarose gel to confirm the amplification of *g2* under the mutagenic conditions, purified using Sap-Exo kit, Jena Bioscience, and cloned into a modified version of pUCP24 plasmid (S1 and S4 Figs). Chemically competent DH5α *E*. *coli* cells were prepared and transformed following the protocol previously reported by Green and Rogers [63]. Around 900 *E*. *coli* colonies were picked and grown overnight in LB-Gm (15 μg/ml). Cultures were mixed in pools of 10 candidates and plasmids were extracted using Wizard Plus SV Minipreps DNA Purification System, Promega, to have pools of plasmids with diverse variants of *g2*, although some empty vectors were also present in the mix. Pools were then electroporated into *P*. *aeruginosa* PA14 following the protocol described by Choi and colleagues [64].

### Selection of *P*. *aeruginosa* candidates carrying *g2* by colony blot

We established a colony blot protocol for detecting the presence of genes in *P*. *aeruginosa* in scale using a radioactive probe (S5 Fig). *P*. *aeruginosa* colonies carrying *g2* (not empty vectors) were selected by colony blot to make the screening more efficient than by PCR. A hundred candidates were streaked on 2 LB-Gm (50 μg/ml) plates (a master plate and a replica plate), along with negative and positive controls (colony with empty plasmid and with *g2*, respectively), and incubated overnight at 37°C. Colonies were transferred from the replica plate to a nylon membrane. Membranes were placed onto filter paper damped with solution I (0.5 M NaOH, 1.5 M NaCl) for 10 min, and then placed onto another filter paper damped with solution II (1 M Tris-HCl (pH 7.2)) for 2 min to neutralise the reaction. Finally, the membranes were placed on the top of filter paper moistened with solution III (0.5 M Tris-HCl, 1.5 M NaCl) for 5 min and exposed to UV light for 5 min in a crosslinker to fix the DNA to the membrane. The membranes were then introduced in hybridisation tubes with 10 ml of 1% SDS, 1 M NaCl solution and incubated at 42°C for 2 h. During this incubation period, an oligonucleotide complementary to *g2* (G2 exp forward, S9 Data) was labelled with Phosphorus-32 following the protocol reported by Novogrodsky and colleagues [65]. The probe was then added to the membranes and incubated at 50°C overnight. The membranes were subsequently washed with 10 ml of 2× SSC for 3 min at room temperature, followed by 2 washes with 2× SSC containing 1% SDS for 5 min each. The membranes were then washed for 30 min with 10 ml of 1× SSC solution, and finally with 10 ml of 0.5× SSC for 15 min. The membranes were allowed to dry before placing them onto an x-ray film in a film cassette. The film was developed, and dark spots on the film produced by the radioactive probe indicated the presence of *g2* in the colony (S5 Fig).

### Phage infection assay

A hundred microliters of overnight cultures of *P*. *aeruginosa* colonies carrying *g2* were mixed with 3.5 ml of TΦ top agar (1% peptone, 0.5% NaCl, 0.7% agar, 10 mM MgSO4) and poured over TΦ plates (1% peptone, 0.5% NaCl, 1.5% agar) containing 50 μg/ml of gentamicin. Serial dilutions of JBD18 phage stock were spotted onto the lawns and the plates were incubated overnight at 37°C (S6 Fig). The EOP was calculated as the titre of the JBD18 phage in PA14 carrying the variant of G2 divided by the titre in PA14 harbouring the WT version of G2. Each infection assay was performed in triplicate. Welch’s *t* test was used and *p*-values were corrected for multiple comparisons using the Bonferroni correction (S5 Data).

### Sequencing and analysis of variants

Colony PCR of the colonies of *P*. *aeruginosa* carrying the variants of *g2* was performed using the oligos MCS pUCP24 forward and MCS pUCP24 reverse (S9 Data). The PCR products were cleaned using the Sap-Exo kit, Jena Bioscience, according to the manufacturer’s specifications and the variants were sequenced using the BigDye Terminator v1.1 Cycle Sequencing Kit, Thermo Fisher Scientific.

### Site-directed mutagenesis

Site-directed mutagenesis of *g2* was done using the Q5 Site-Directed Mutagenesis Kit, New England Biolabs, according to the manufacturer’s specifications, using the NEBaseChanger tool (http://nebasechanger.neb.com/) for oligo design. To generate multiple changes of amino acids in a single position, the primers were designed with 1 or 2 random nucleotides in the codon of the target residue (S9 Data, primers V40, D29, and Y32).

### dN/dS analysis

An analysis to determine the dN/dS ratio for each codon was performed using codeml from the PAML package v4.9 [66] on 130 *acrIF7* sequences, including *g2* and the genes encoding the proteins of the subclusters sc1 and sc2, but excluding GCF_900707915-WP_134600237.1 because the sequence is shorter in the 3′ end and therefore would introduce gaps in the alignment which are not tolerated by the program. From those, 119 sequences belonged to sc1 and 11 to subcluster sc2. We focused on sc1 and sc2 because they represent the predominant subclusters, accounting for around 90% of all the members in the AcrIF7 family. Additionally, the sequences from the other subclusters are too divergent in the nucleotide level, and therefore, would compromise the reliability of the results. The nucleotide alignment was trimmed in the 5′ end to the length of *g2* to have an alignment of the core codons (S10 Data). The tree used for the analysis was obtained from IQTree v1.3.11 [67], with the model K2+G4. We fitted the data to the site models M1a (NearlyNeutral) and M2a (PositiveSelection) and performed a likelihood ratio χ2 test of both models. Finally, Empirical Bayes (EB) [66] was used to calculate the posterior probabilities for site classes and identify dN/dS values for each codon (S6 Data).

### Protein modelling and docking analyses

G2 WT and mutants were modelled using AlphaFold2 [33]. Analysis of the models was done using open-source Pymol (Schrodinger, LLC, 2010, the PyMOL Molecular Graphics System, Version 2.4.0). The binding position of G2 or its mutants on Cas8f (PDB code 7JZX and chain A) was predicted using HADDOCK 2.4 [68] (S14 Data), as described by Kim and colleagues [18]. The residue–residue interactions of G2/mutants and Cas8f were analysed using Ligplot+ [69]. The secondary structure alignment of G2 and its mutants was visualised using 2dSS [70], and analysis of the models and superpositions was done using chimeraX [71].

### Experimental evolution of phage H70

Evolution experiments were performed by mixing an ancestral (initial) H70 phage stock and overnight cultures of either PA14 WT or PA14 ΔCR in 30 ml of fresh LB to a final concentration of 10^6^ PFU/ml and 10^6^ CFU/ml. Cultures were incubated for 16 h at 37°C with shaking and then centrifuged at 4,000 rpm for 12 min. Supernatants containing the evolved phage were filter-sterilised and titred on both PA14 and PA14ΔCR, irrespective of where they were propagated. The consequent passages (10 in total, 3 lineages each) were done by mixing the evolved phage stock with fresh overnight cultures of the same strain used in the first passage (PA14 or PA14ΔCR) to a final concentration of 10^6^ PFU/ml and 10^6^ CFU/ml followed by the same steps as in passage 1. Each titration assay was performed in triplicate and the EOP were calculated using PA14 ΔCR as reference (titre on PA14/ titre on PA14ΔCR) (Table A in S7 Data). *P*-values were calculated using a one-way ANOVA test on each dataset. DNA from samples from passage 10 was extracted and combined in 1 sample per strain (H70 evolved in PA14 WT and H70 evolved in PA14 ΔCR) for Illumina sequencing. Reads were mapped against the parental strain (accession number NC_027384.1) using BWA mem v0.7.17-r1188 [72] and variants were identified using pilon v1.22 with the default settings for variant calling. The percentage of each nucleotide in each position of the H70 genome was calculated based on the number of reads with that particular nucleotide and the total number of reads covering that position. The data were filtered to exclude the variants with the reference nucleotide and those representing less than 1% of the population (Table B–D in S7 Data).

## Supporting information

S1 Fig Modified version of the expression vector pUCP24. The vector map shows a modified version of the pUCP24 plasmid (named pCUP24-L3). The changes made to the original plasmid sequence are indicated in light green boxes. The yellow (lacZα) and green (*g2*) arrows represent the coding regions in the MCS of the plasmids. The changes in the plasmid involved moving the EcoRI restriction site in pUCP24 upstream to prevent the incorporation of additional amino acids from lacZα peptide into G2 once it is cloned.
(DOCX)

S2 Fig Phage prediction and comparison of representative prophage and phage-like sequences from NC2. (A) An example of the results obtained with the phage prediction program PHASTER using a putative prophage sequence identified from a flanking region in NC2 (see Fig 4; results for all regions are provided in Table A in S3 Data). (B) Comparison at the nucleotide level of 4 prophage sequences identified from analysis of flanking regions in NC2 and phage H70. Functions of the reference sequence in the comparison (GCF_000510305, innermost ring) are indicated at the centre of the figure. Coordinates of the identified prophage sequences are provided in Table B in S3 Data.
(DOCX)

S3 Fig Diversity of AcrIF7, Aca1, and Cas8f homologs encoded in *P*. *aeruginosa* and *Pseudomonas* phage genomes. The neighbour-joining trees in circular (top) and unrooted (bottom) format illustrate the comparison of AcrIF7, Aca1, and Cas8f homologs identified through BLASTp searches against proteins encoded in *P*. *aeruginosa* and *Pseudomonas* phage genomes. The total number of compared sequences (aligned with the MUSCLE algorithm) is indicated on the left side of the circular representation of the trees. Instances where AcrIF7 was identified in the same genome as Aca1/Cas8f are highlighted in green in both the circular and unrooted trees. Purple dots on tree branches represent bootstrap support values >80 calculated from 100 replicates. The bar plots and sequence logos shown at the bottom of the figure represent the conservation level and consensus sequence determined for each of the 3 proteins from the alignment of nonredundant sequences. The number of nonredundant sequences in the alignment is indicted on the left side of the bar plots, along with the alignment length. The average conservation of the proteins (see Methods and Table D in S8 Data) is indicated on the right side. The alignment of AcrIF7, Aca1 and Cas8f homologs, and the genome and protein accessions, are provided as Tables A–C in S8 Data and S11–S13 Data.
(DOCX)

S4 Fig Strategy for cloning and identification of G2 mutants. The figure illustrates the steps followed to generate the collection of G2 random mutants presented in this study. The strategy consisted of (1) cloning the error-prone PCR products into pUCP24-L3; (2) transformation and extraction of pools of plasmids from *E*. *coli*; (3) electroporation of the pools into *Pseudomonas aeruginosa* PA14; (4) assessment of the efficiency of the variant to block the CRISPR-Cas system; and (5) sequencing of the mutants and analysis of the protein model. Figure created with BioRender.com.
(DOCX)

S5 Fig Colony blot protocol. (I) The protocol consisted of streaking candidates in 2 LB plates (master and replica plates), followed by transferring the colonies to a nylon membrane. The membrane is processed with different solutions (see Methods) and hybridised with a radioactive probe. X-ray films are exposed to the membranes and developed to identify colonies with *g2*. (II) The lower panel shows the x-ray films from 7 different membranes. Black spots confirm the presence of g2 in the colonies, which are then identified based on the position in the membrane (with the help of a grid template that was also used when streaking the colonies-H). The white arrows point to the positive controls included in each membrane.
(DOCX)

S6 Fig G2-phenotyping assay. The activity of G2 was assessed based on the ability of the variant to block the CRISPR-cas system and allow the infection of a CRISPR-sensitive phage. In Fig 4, different scenarios are shown: (A) PA14 with an active CRISPR-cas system that blocks the infection by a CRISPR-sensitive phage (SP), (B) PA14 ΔCR mutant that can no longer defend against SP, (C) PA14 WT transformed with a wild-type version of the anti-CRISPR G2, which inhibits the CRISPR-cas system and therefore allows the infection the SP, and (D) PA14 WT carrying mutant versions of G2 that are defective at suppressing the CRISPR-cas system, hence SP cannot infect the cell. The left panel shows the different scenarios at the cellular level and the right panel illustrates the phenotypes seen in bacterial lawns.
(DOCX)

S7 Fig The secondary structures of AcrIF7 variants. The secondary structure of G2 mutants was predicted using 2dSS. The beta sheets are represented in the figure as yellow arrows, while the alpha helices are represented as black wavy lines.
(DOCX)

S1 Data *Pseudomonas aeruginosa* genomes carrying AcrIF7 and associated metadata. (XLSX)

S2 Data AcrIF7 homologs similarity. Table A. AcrIF7 homologs similarity. Table B. Unique AcrIF7 homologs occurrences.
(XLSX)

S3 Data AcrIF7 flaking regions. Table A. Flanking regions of AcrIF7 in diverse genomes. Table B. Diverse prophage regions carrying AcrIF7.
(XLSX)

S4 Data Random mutagenesis conditions. (XLSX)

S5 Data EOP of G2 mutants. (XLSX)

S6 Data Omega (dN/dS) values for AcrIF7 homologs. (XLSX)

S7 Data Experimental evolution: EOP of evolved populations and mutations found. Table A. Experimental evolution EOP. Table B. Experimental evolution unique mutations in phage population passaged in PA14 WT. Table C. Experimental evolution unique mutations in phage population passaged in PA14 dCR. Table D. Experimental evolution shared mutations present in both phage populations.
(XLSX)

S8 Data Diversity of AcrIF7, Aca1, and Cas8f in *Pseudomonas aeruginosa* genomes. Table A. *Pseudomonas aeruginosa* AcrIf7 diversity. Table B. *Pseudomonas aeruginosa* Cas8f diversity. Table C. *Pseudomonas aeruginosa* Cas8f diversity. Table D. Average conservation of *Pseudomonas aeruginosa* AcrIF7, Aca1, and Cas8f.
(XLSX)

S9 Data Primer sequences. (XLSX)

S10 Data Alignment of AcrIF7 homologs used as input for dN/dS analysis in codeml. Please open with a text editor.
(FST)

S11 Data Alignment of AcrIF7 homologs used in diversity analysis. Please open with a text editor.
(FST)

S12 Data Alignment of Cas8f homologs used in diversity analysis. Please open with a text editor.
(FST)

S13 Data Alignment of Aca1 homologs used in diversity analysis. Please open with a text editor.
(FST)

S14 Data Docking files from protein–protein interaction analysis. Individual files can be open using Pymol or any structure visualisation tool.
(ZIP)

## Abbreviations

CRISPR: clustered regularly interspaced short palindromic repeats
EOP: efficiency of plating
MLST: multilocus sequence typing
NC1: network cluster 1
RGP: region of genomic plasticity
WT: wild-type
