# Supplementary material for: Distribution and molecular evolution of the anti-CRISPR family AcrIF7
Source: PLoS Biol. 2023 Apr 21;21(4):e3002072. doi: 10.1371/journal.pbio.3002072 (PMC10155984; doi:10.1371/journal.pbio.3002072)
Supplement: S1 Fig — The vector map shows a modified version of the pUCP24 plasmid (named pCUP24-L3). The changes made to the original plasmid sequence are indicated in light green boxes. The yellow (lacZα) and green (g2) arrows represent the coding regions in the MCS of the plasmids. The changes in the plasmid involved moving the EcoRI restriction site in pUCP24 upstream to prevent the incorporation of additional amino acids from lacZα peptide into G2 once it is cloned. (DOCX) [file pbio.3002072.s001.docx]

**
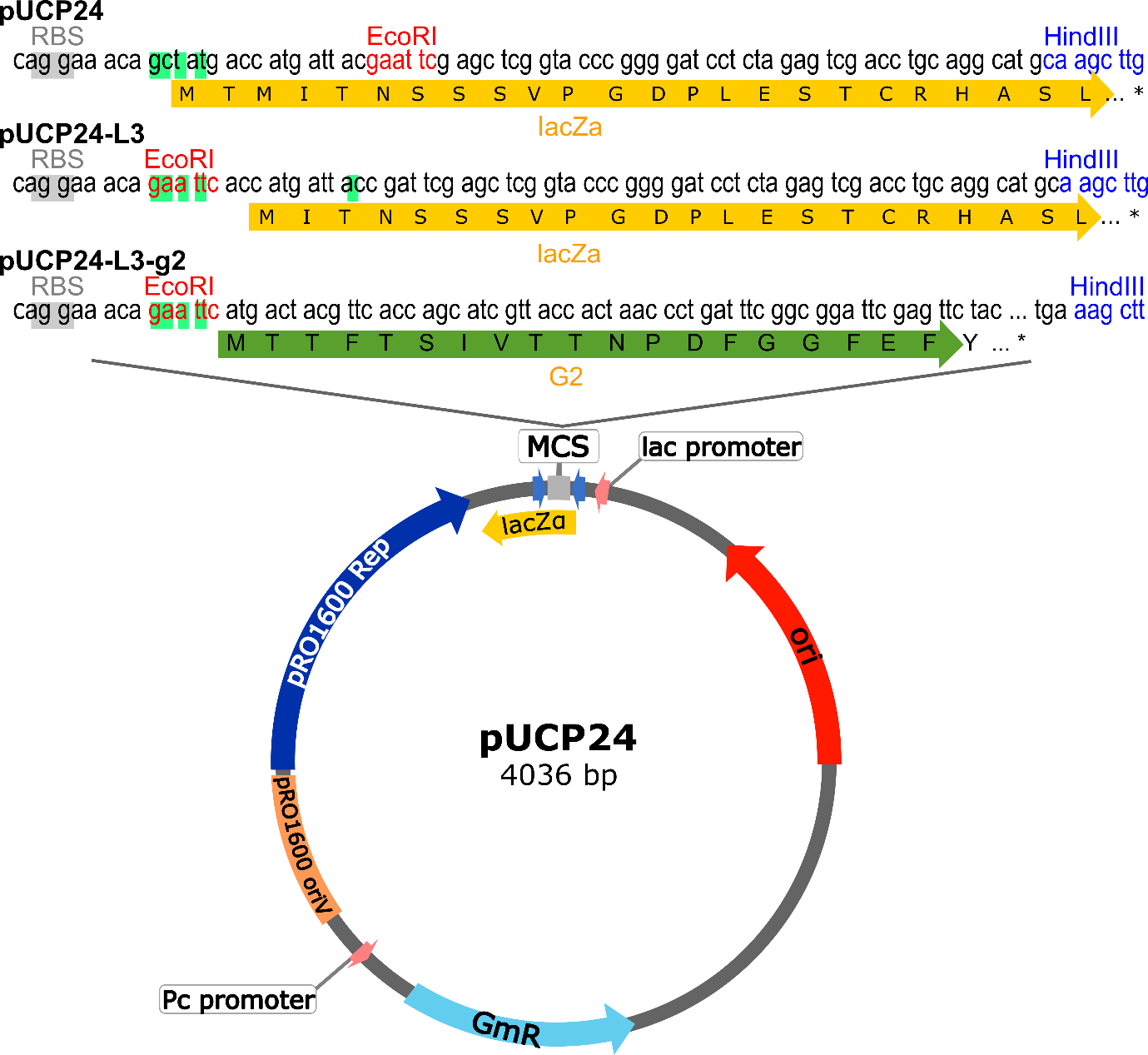
**

**S1 Fig. Modified version of the expression vector pUCP24.** The vector map shows a modified version of the pUCP24 plasmid (named pCUP24-L3). The changes made to the original plasmid sequence are indicated in light green boxes. The yellow (lacZα) and green (*g2*) arrows represent the coding regions in the MCS of the plasmids. The changes in the plasmid involved moving the EcoRI restriction site in pUCP24 upstream to prevent the incorporation of additional amino acids from lacZα peptide into G2 once it is cloned.
