# Supplementary material for: Distribution and molecular evolution of the anti-CRISPR family AcrIF7
Source: PLoS Biol. 2023 Apr 21;21(4):e3002072. doi: 10.1371/journal.pbio.3002072 (PMC10155984; doi:10.1371/journal.pbio.3002072)
Supplement: S2 Fig — (A) An example of the results obtained with the phage prediction program PHASTER using a putative prophage sequence identified from a flanking region in NC2 (see Fig 4; results for all regions are provided in Table A in S3 Data). (B) Comparison at the nucleotide level of 4 prophage sequences identified from analysis of flanking regions in NC2 and phage H70. Functions of the reference sequence in the comparison (GCF_000510305, innermost ring) are indicated at the centre of the figure. Coordinates of the identified prophage sequences are provided in Table B in S3 Data. (DOCX) [file pbio.3002072.s002.docx]

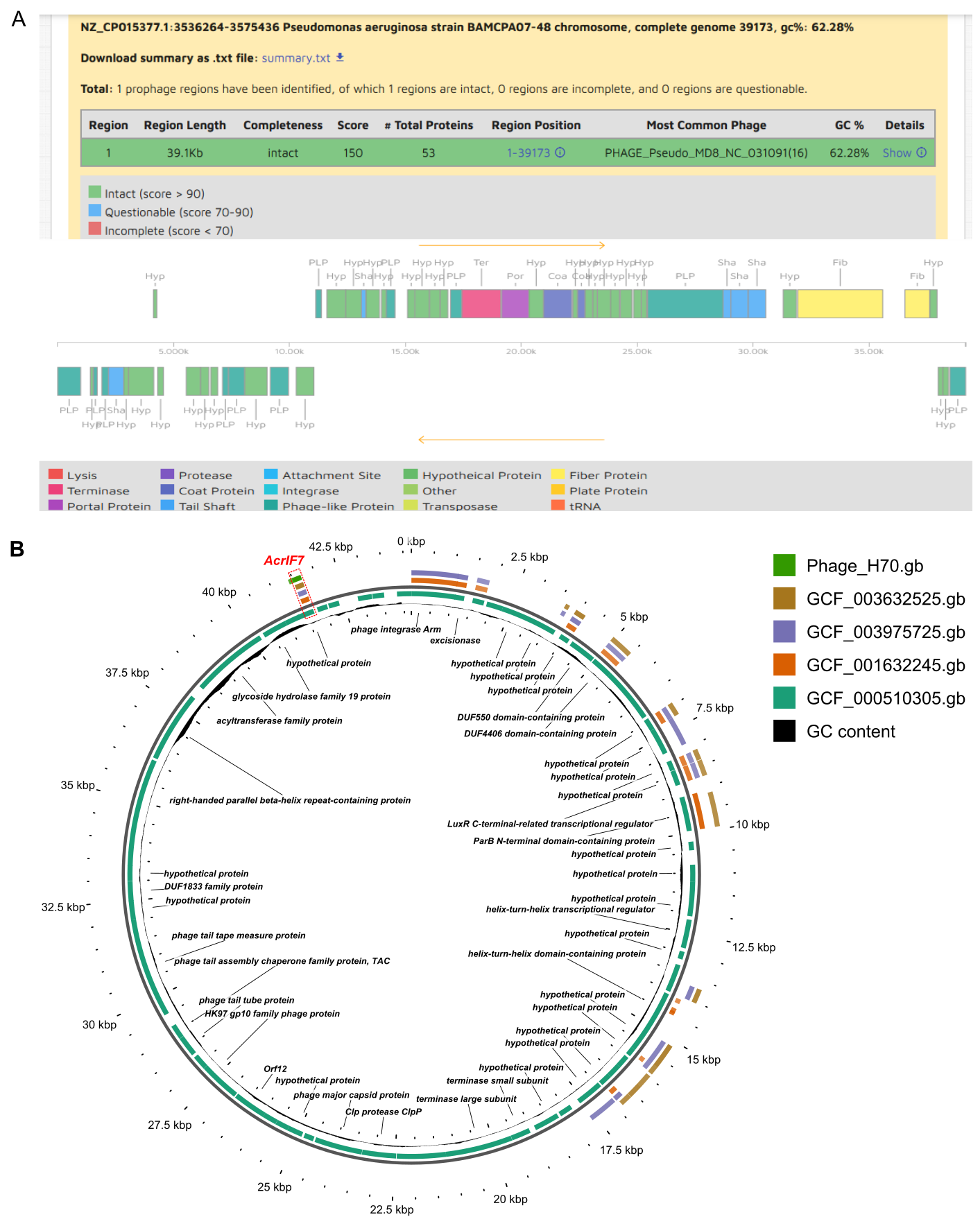


**S2 Fig. Phage prediction and comparison of representative prophage and phage-like sequences from NC2**. A) An example of the results obtained with the phage prediction program PHASTER using a putative prophage sequence identified from a flanking region in NC2 (See Figure 4. Results for all regions are provided in S3 Data). B) Comparison at the nucleotide level of 4 prophage sequences identified from analysis of flanking regions in NC2, and phage H70. Functions of the reference sequence in the comparison (GCF_000510305, innermost ring) are indicated at the centre of the figure. Coordinates of the identified prophage sequences are provided in S3 Data.
