# Supplementary material for: Distribution and molecular evolution of the anti-CRISPR family AcrIF7
Source: PLoS Biol. 2023 Apr 21;21(4):e3002072. doi: 10.1371/journal.pbio.3002072 (PMC10155984; doi:10.1371/journal.pbio.3002072)
Supplement: S3 Fig — The neighbour-joining trees in circular (top) and unrooted (bottom) format illustrate the comparison of AcrIF7, Aca1, and Cas8f homologs identified through BLASTp searches against proteins encoded in P. aeruginosa and Pseudomonas phage genomes. The total number of compared sequences (aligned with the MUSCLE algorithm) is indicated on the left side of the circular representation of the trees. Instances where AcrIF7 was identified in the same genome as Aca1/Cas8f are highlighted in green in both the circular and unrooted trees. Purple dots on tree branches represent bootstrap support values >80 calculated from 100 replicates. The bar plots and sequence logos shown at the bottom of the figure represent the conservation level and consensus sequence determined for each of the 3 proteins from the alignment of nonredundant sequences. The number of nonredundant sequences in the alignment is indicted on the left side of the bar plots, along with the alignment length. The average conservation of the proteins (see Methods and Table D in S8 Data) is indicated on the right side. The alignment of AcrIF7, Aca1 and Cas8f homologs, and the genome and protein accessions, are provided as Tables A–C in S8 Data and S11–S13 Data. (DOCX) [file pbio.3002072.s003.docx]

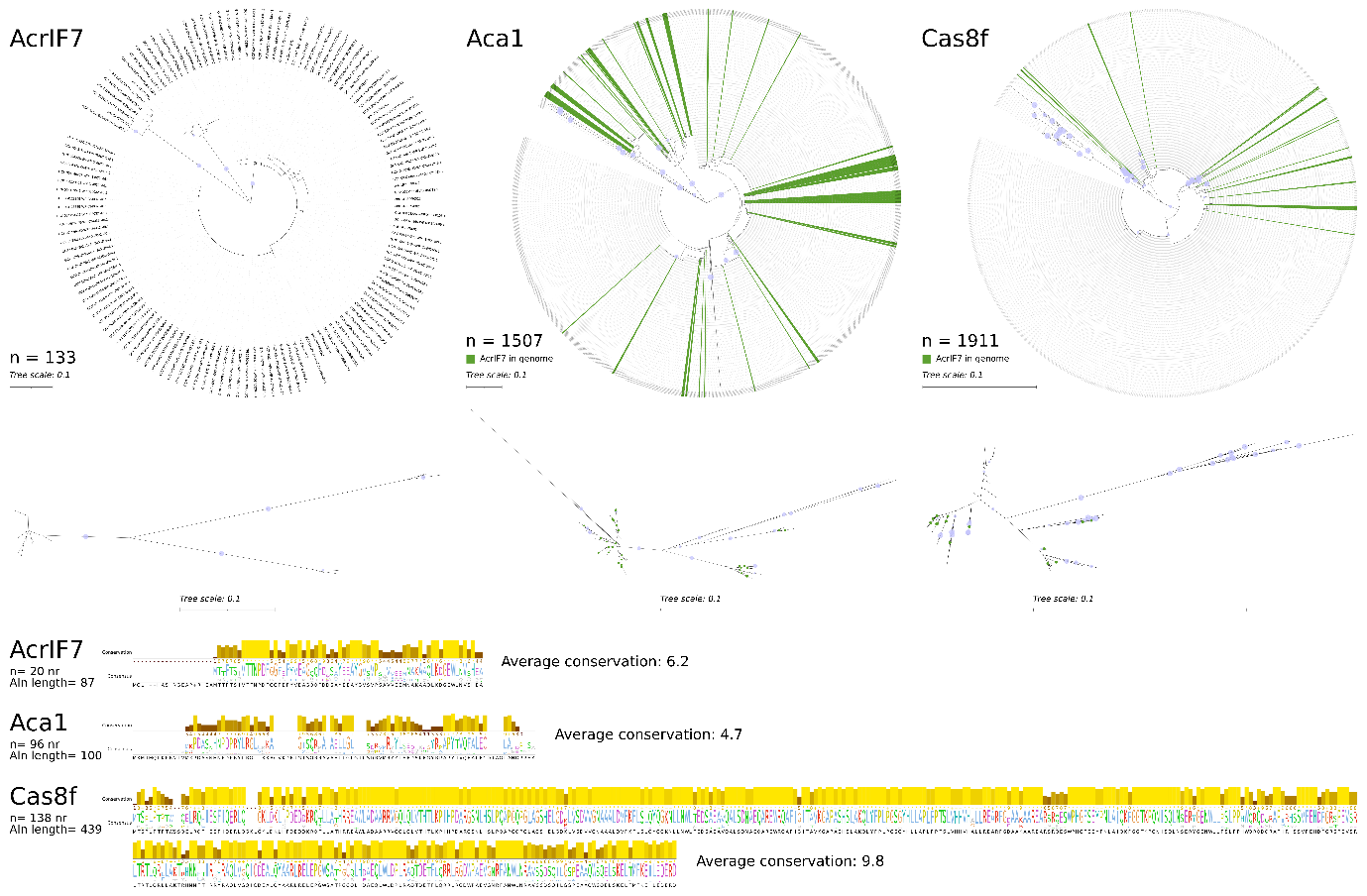


**S3 Fig. Diversity of AcrIF7, Aca1 and Cas8f homologs encoded in *P. aeruginosa* and *Pseudomonas* phage genomes.** The neighbor-joining trees in circular (top) and unrooted (bottom) format illustrate the comparison of AcrIF7, Aca1 and Cas8f homologs identified through BLASTp searches against proteins ecoded in *P. aeruginosa* and *Pseudomonas* phage genomes. The total number of compared sequences (aligned with the MUSCLE algorithm) is indicated on the left side of the circular representation of the trees. Instances where AcrIF7 was identified in the same genome as Aca1/Cas8f are highlighted in green in both the circular and unrooted trees. Purple dots on tree branches represent bootstrap support values >80 calculated from 100 replicates. The bar plots and sequence logos shown at the bottom of the figure represent the conservation level and consensus sequence determined for each of the three proteins from the alignment of non-redundant sequences. The number of non-redundant sequences in the alignment is indicted on the left side of the bar plots, along with the alignment length. The average conservation of the proteins (see Methods and S8 Data) is indicated on the right side. The alignment of AcrIF7, Aca1 and Cas8f homologs, and the genome and protein accessions, are provided as S8 Data and S11-13 Data.
