# Supplementary material for: Distribution and molecular evolution of the anti-CRISPR family AcrIF7
Source: PLoS Biol. 2023 Apr 21;21(4):e3002072. doi: 10.1371/journal.pbio.3002072 (PMC10155984; doi:10.1371/journal.pbio.3002072)
Supplement: S4 Fig — The figure illustrates the steps followed to generate the collection of G2 random mutants presented in this study. The strategy consisted of (1) cloning the error-prone PCR products into pUCP24-L3; (2) transformation and extraction of pools of plasmids from E. coli; (3) electroporation of the pools into Pseudomonas aeruginosa PA14; (4) assessment of the efficiency of the variant to block the CRISPR-Cas system; and (5) sequencing of the mutants and analysis of the protein model. Figure created with BioRender.com. (DOCX) [file pbio.3002072.s004.docx]

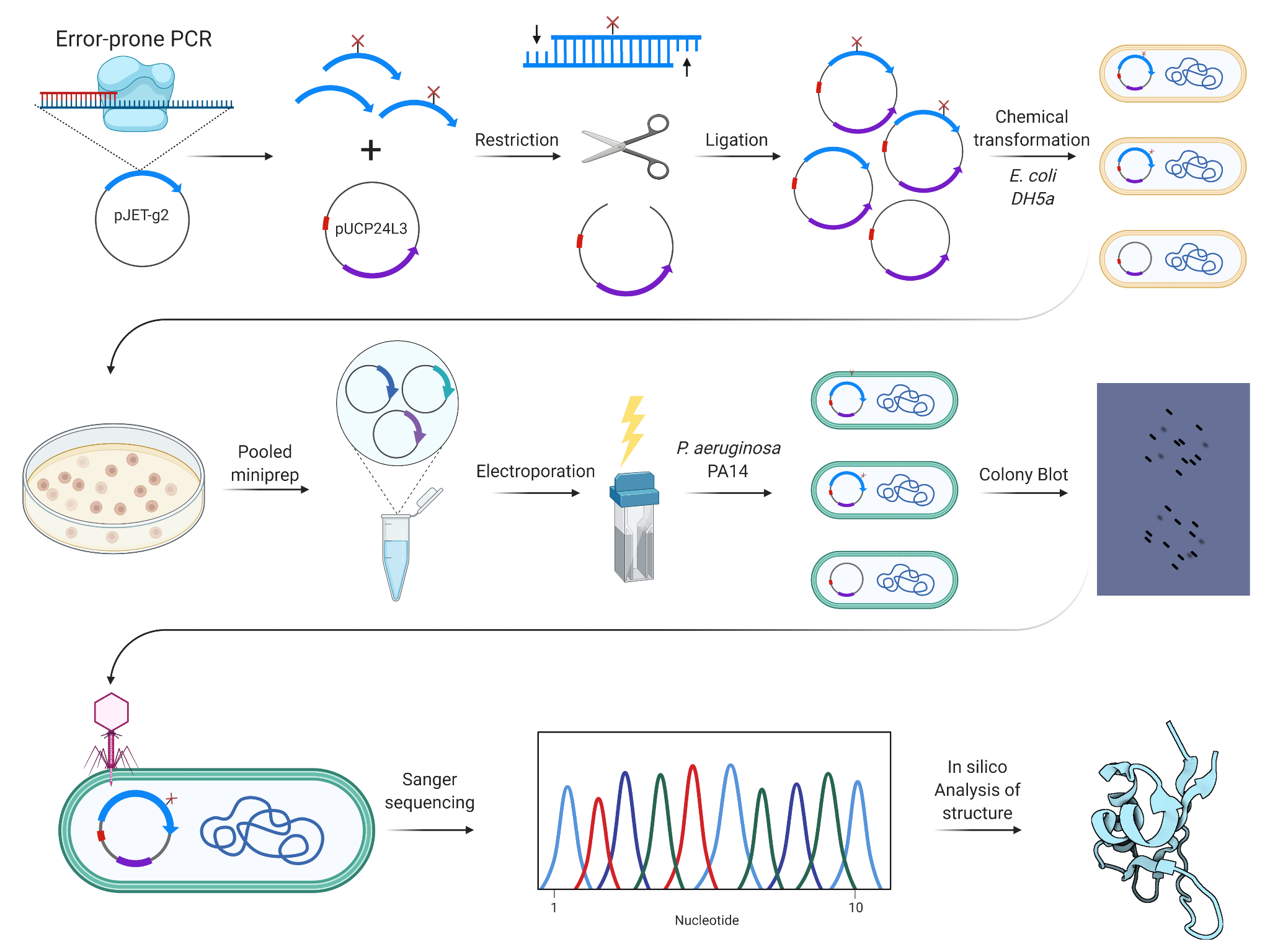


**S4 Fig. Strategy for cloning and identification of G2 mutants.** The figure illustrates the steps followed to generate the collection of G2 random mutants presented in this study. The strategy consisted of 1) cloning the error-prone PCR products into pUCP24-L3, 2) transformation and extraction of pools of plasmids from *E. coli*, 3) electroporation of the pools into *Pseudomonas aeruginosa* PA14, 4) assessment of the efficiency of the variant to block the CRISPR-Cas system and 5) sequencing of the mutants and analysis of the protein model. Figure created with BioRender.com.
