# Supplementary material for: Distribution and molecular evolution of the anti-CRISPR family AcrIF7
Source: PLoS Biol. 2023 Apr 21;21(4):e3002072. doi: 10.1371/journal.pbio.3002072 (PMC10155984; doi:10.1371/journal.pbio.3002072)
Supplement: S5 Fig — (I) The protocol consisted of streaking candidates in 2 LB plates (master and replica plates), followed by transferring the colonies to a nylon membrane. The membrane is processed with different solutions (see Methods) and hybridised with a radioactive probe. X-ray films are exposed to the membranes and developed to identify colonies with g2. (II) The lower panel shows the x-ray films from 7 different membranes. Black spots confirm the presence of g2 in the colonies, which are then identified based on the position in the membrane (with the help of a grid template that was also used when streaking the colonies-H). The white arrows point to the positive controls included in each membrane. (DOCX) [file pbio.3002072.s005.docx]

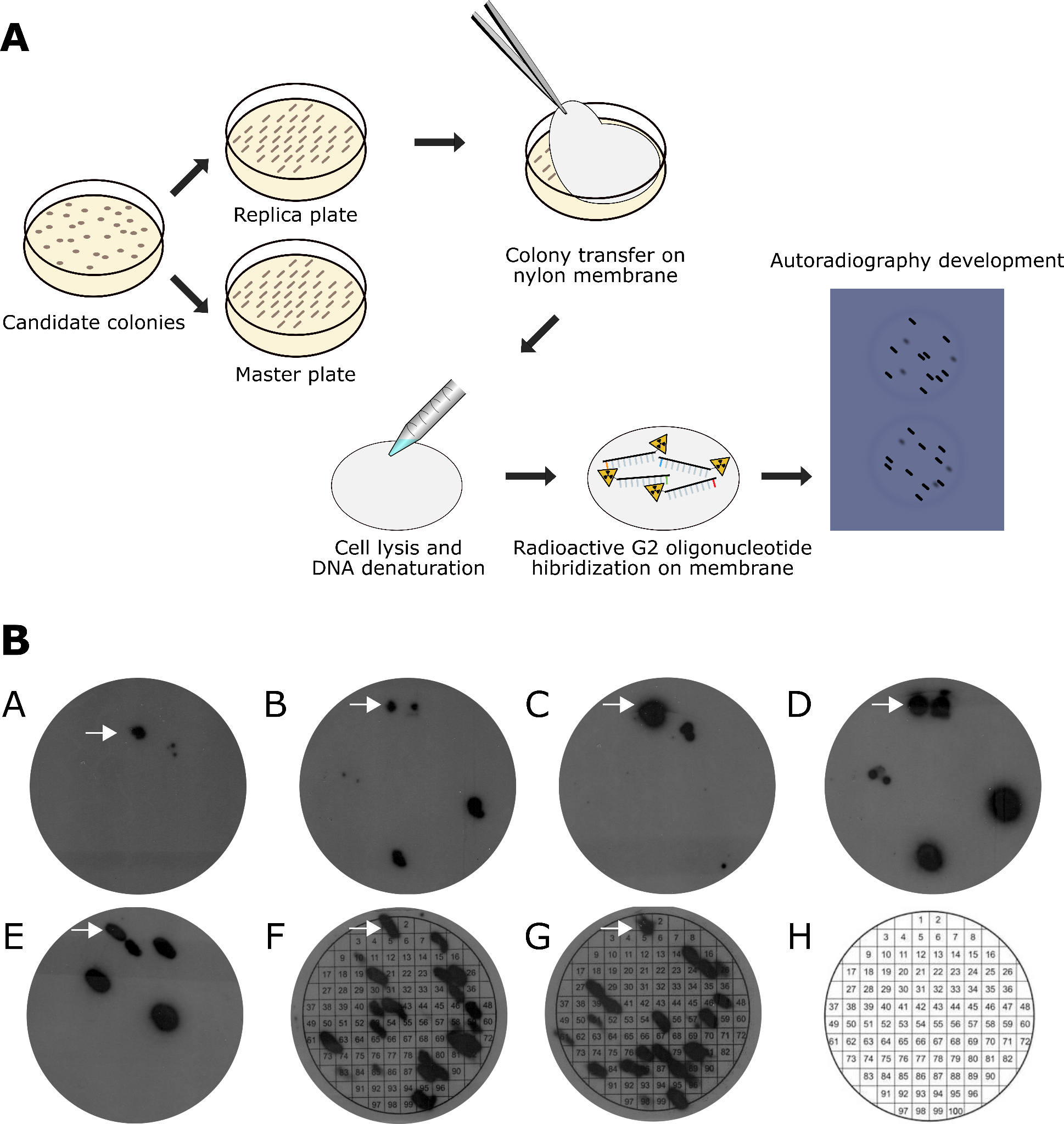


**S5 Fig. Colony blot protocol.** I) The protocol consisted of streaking candidates in 2 LB plates (master and replica plates), followed by transferring the colonies to a nylon membrane. The membrane is processed with different solutions (see Methods) and hybridised with a radioactive probe. X-ray films are exposed to the membranes and developed to identify colonies with *g2*. II) The lower panel shows the x-ray films from 7 different membranes. Black spots confirm the presence of g2 in the colonies, which are then identified based on the position in the membrane (with the help of a grid template that was also used when streaking the colonies-H). The white arrows point to the positive controls included in each membrane.
