# Supplementary material for: Distribution and molecular evolution of the anti-CRISPR family AcrIF7
Source: PLoS Biol. 2023 Apr 21;21(4):e3002072. doi: 10.1371/journal.pbio.3002072 (PMC10155984; doi:10.1371/journal.pbio.3002072)
Supplement: S6 Fig — The activity of G2 was assessed based on the ability of the variant to block the CRISPR-cas system and allow the infection of a CRISPR-sensitive phage. In Fig 4, different scenarios are shown: (A) PA14 with an active CRISPR-cas system that blocks the infection by a CRISPR-sensitive phage (SP), (B) PA14 ΔCR mutant that can no longer defend against SP, (C) PA14 WT transformed with a wild-type version of the anti-CRISPR G2, which inhibits the CRISPR-cas system and therefore allows the infection the SP, and (D) PA14 WT carrying mutant versions of G2 that are defective at suppressing the CRISPR-cas system, hence SP cannot infect the cell. The left panel shows the different scenarios at the cellular level and the right panel illustrates the phenotypes seen in bacterial lawns. (DOCX) [file pbio.3002072.s006.docx]

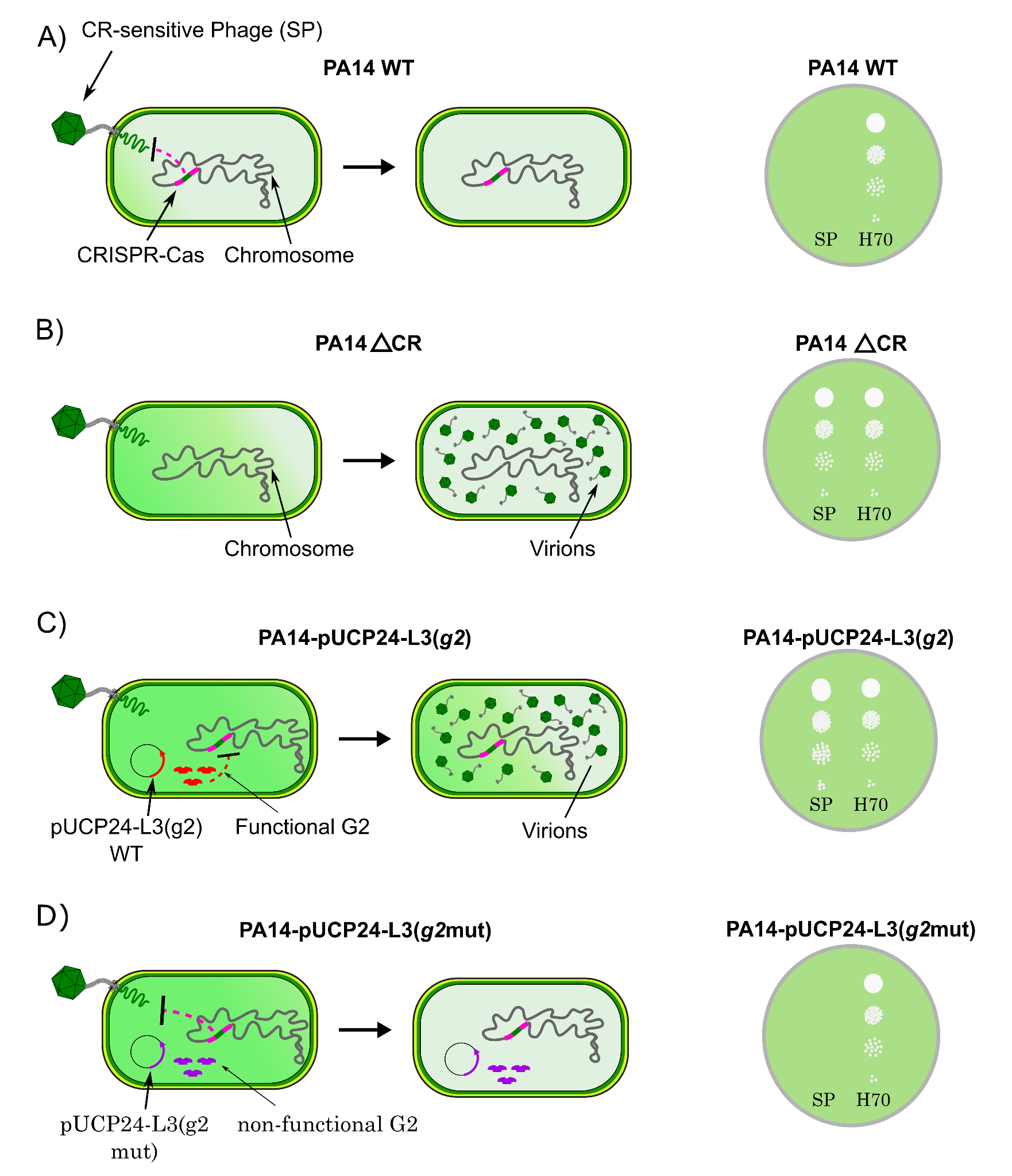


**S6 Fig. G2-phenotyping assay.** The activity of G2 was assessed based on the ability of the variant to block the CRISPR-cas system and allow the infection of a CRISPR-sensitive phage. In figure 4 different scenarios are shown: A) PA14 with an active CRISPR-cas system that blocks the infection by a CRISPR-sensitive phage (SP), B) PA14 ΔCR mutant that can no longer defend against SP C) PA14 WT transformed with a wild-type version of the anti-CRISPR G2, which inhibits the CRISPR-cas system and therefore allows the infection the SP, and D) PA14 WT carrying mutant versions of G2 that are defective at suppressing the CRISPR-cas system, hence SP cannot infect the cell. The left panel shows the different scenarios at the cellular level and the right panel illustrates the phenotypes seen in bacterial lawns.
