# Supplementary material for: Distribution and molecular evolution of the anti-CRISPR family AcrIF7
Source: PLoS Biol. 2023 Apr 21;21(4):e3002072. doi: 10.1371/journal.pbio.3002072 (PMC10155984; doi:10.1371/journal.pbio.3002072)
Supplement: S7 Fig — The secondary structure of G2 mutants was predicted using 2dSS. The beta sheets are represented in the figure as yellow arrows, while the alpha helices are represented as black wavy lines. (DOCX) [file pbio.3002072.s007.docx]

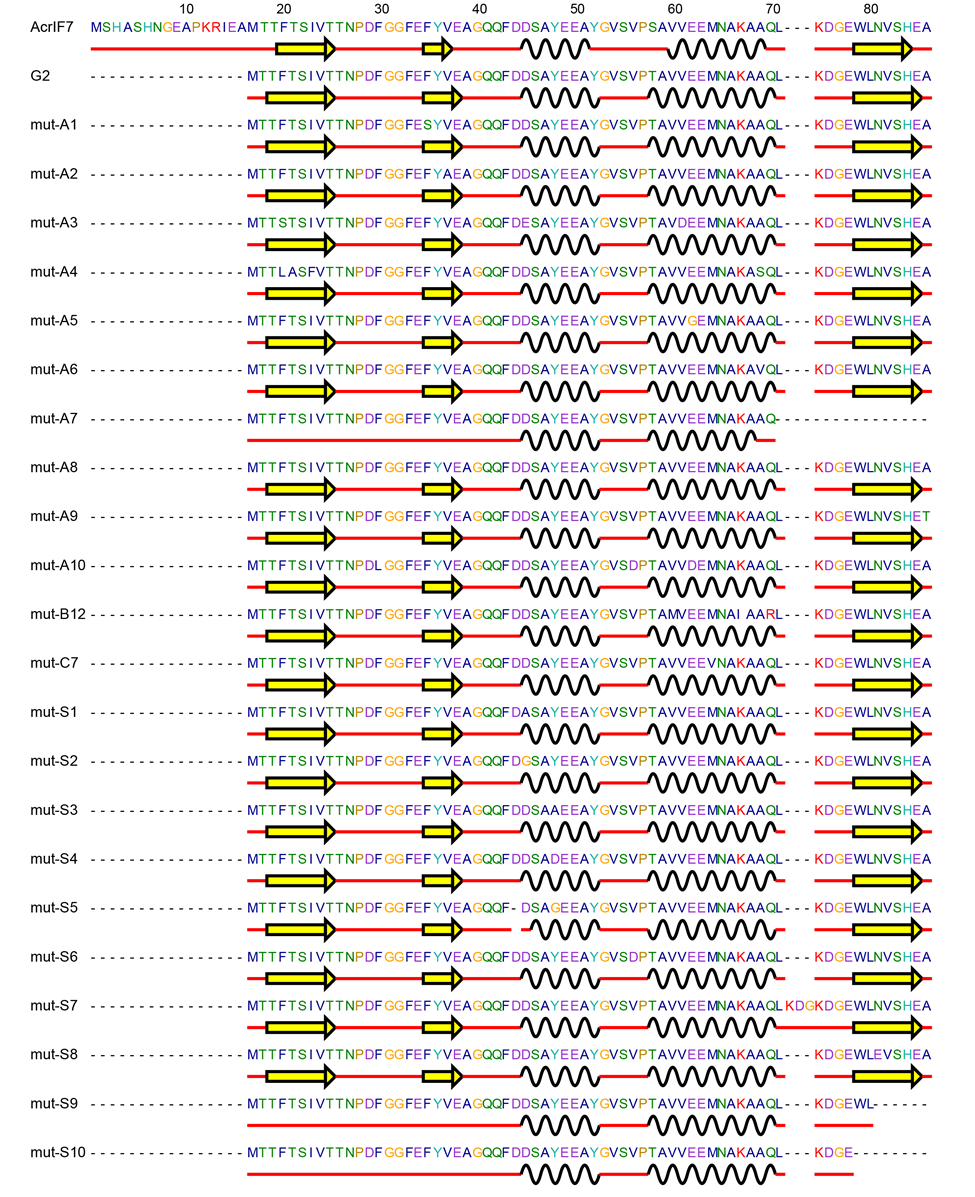


**S7 Fig. The secondary structures of AcrIF7 variants.** The secondary structure of G2 mutants was predicted using 2dSS. The beta-sheets are represented in the figure as yellow arrows, while the alpha helices are represented as black wavy lines.
